# Targeting Intratumoral Bacteria for Enhanced Tumor Suppression with Nano-Based Therapeutics: A Scoping Review

**DOI:** 10.3390/pharmaceutics18030318

**Published:** 2026-03-02

**Authors:** Tianxiang Yi, Zhiyou Dong, Sharon Shui Yee Leung

**Affiliations:** 1 School of Pharmacy, Faculty of Medicine, The Chinese University of Hong Kong, Sha Tin, New Territories, Hong Kong SAR, China; yitx@link.cuhk.edu.hk (T.Y.); zhiyoudong@cuhk.edu.hk (Z.D.); 2 Guangdong–Hong Kong–Macao Joint Laboratory for New Drug Screening, School of Pharmacy, The Chinese University of Hong Kong, Sha Tin, New Territories, Hong Kong SAR, China

**Keywords:** intratumoral bacteria, bacteria-induced chemoresistance, protumor bacteria, nanomedicines, immunomodulation, precision oncology

## Abstract

**Background**: Increasing evidence identifies intratumoral bacteria as key modulators of tumor progression, chemoresistance, and immunosuppression, presenting major obstacles to conventional cancer therapies. Recent advances in nanotechnology have enabled new strategies for selective targeting bacteria within the tumor microenvironment, potentially improving anticancer efficacy. **Methods**: A scoping review was conducted to outline the current landscape of nano-based therapeutic approaches aimed at the simultaneous elimination of intratumoral bacteria and cancer. Preclinical research publications involving in vivo antitumor efficacy evaluations were retrieved from three databases, Web of Science, PubMed, and Scopus, using the key words “(kill* OR eradicate* OR eliminate*) AND intratumoral AND (bacteria OR infection)”. Key information from the eligible studies was extracted and analyzed. **Results**: The diversity of bacterial species, cancer models, and evaluation methodologies employed in these preclinical studies were summarized, followed by critical examination of the design principles, therapeutic outcomes, and translational challenges of various nanomedicine platforms, including passive and active targeting drug delivery systems, phototherapy, phage therapy, and emerging modalities. Nano-based therapeutics functionalized with both antibacterial and anticancer properties were shown to effectively overcome bacteria-induced treatment resistance. **Conclusions**: Targeting intratumoral bacteria may significantly enhance the efficacy of existing treatments and contribute to the evolution of precision oncology. The insights gained from this review are expected to guide future systematic reviews and inform research directions in the development of dual-functional nanomedicines for cancer therapy.

## 1. Introduction

Malignancy has been a major global public health concern due to its high incidence and mortality [1]. Multiple factors contribute to tumor initiation and progression, including UV radiation, chronic inflammation, and infections [2,3]. Traditionally, tumor tissues were considered sterile spaces. However, increasing evidence indicates that microorganisms, which play critical roles in human health, can reside within tumor tissues, with microbial compositions markedly different from those in normal tissues [4]. Indeed, microbiota dysbiosis has recently been reported to be strongly associated with carcinogenesis and tumor progression. Certain species contribute to tumor initiation and promotion by driving chronic inflammation, inducing DNA damage, modulating immune evasion, reprogramming metabolism, or activating oncogenic signaling pathways [4,5]. For instance, *Fusobacterium nucleatum* is prominently associated with colorectal, breast, pancreatic, head and neck cancers, where it drives chronic inflammation via TLR4/MyD88/NF-κB signaling, promotes adhesion and invasion through FadA and Fap2 proteins, activates Wnt/β-catenin pathways, and excludes T cells from the tumor microenvironment. Intratumoral microbiota may even impair the efficacy of anticancer treatments [6]. For example, *F. nucleatum* induces autophagy (upregulation of LC3-II) and NF-κB/BIRC3-mediated anti-apoptotic effects, reducing sensitivity to 5-fluorouracil (5-FU), oxaliplatin (OXA), or gemcitabine [7]. In contrast, pks^+^ *Escherichia coli* is mainly implicated in colorectal and pancreatic cancers, where it produces the genotoxin colibactin, causing DNA double-stranded breaks that contribute to tumor initiation. It also expresses the long isoform of cytidine deaminase, which metabolizes gemcitabine to its inactive form (2′,2′-difluorodeoxyuridine, dFdU), thereby conferring chemoresistance [8]. Similarly, enterotoxigenic *Bacteroides fragilis* plays a role in colorectal and pancreatic cancers by secreting the toxin BFT, which triggers IL-17/STAT3/NF-κB-driven chronic inflammation and epithelial barrier disruption during initiation and progression, while STAT3 activation upregulates anti-apoptotic genes and inflammatory cytokines that promote resistance to gemcitabine or irinotecan [9,10]. These context-specific contributions highlight the heterogeneity of intratumoral microbiota effects on tumor progression.

Furthermore, the permissive and immune-evasive nature of the tumor microenvironment (TME) provides a unique shelter for pathogenic bacteria to escape host immune surveillance and antibacterial defense, contributing to recurrent infections and poor disease prognosis in cancer patients [5]. The empirical use of antibiotics in cancer patients has long been a common practice to treat and prevent bacterial infections. However, recent epidemiological studies have indicated that repeated exposure to broad-spectrum antibiotics may lead to several adverse outcomes, including (i) serious side effects due to antibiotic toxicity [11]; (ii) impaired anticancer efficacy due to antagonistic interactions between drugs and/or flora imbalance [12]; and (iii) emergence of antibiotic-resistant strains [13]. A previous study showed that pre-resection antibiotic treatment targeting anaerobic bacteria could improve disease-free survival in colorectal cancer (CRC) patients by 25.5% [14]. This promising observation suggests that the precise delivery of antibiotics to target bacteria residing within tumor tissues could be an effective strategy in overcoming the limitations of current antibacterial management in cancer patients.

Leveraging the enhanced permeability and retention (EPR) effect due to the leaky vasculature within tumor regions, nanoparticles can effectively accumulate in tumor tissues [15]. Nanotechnology-based formulations, like liposomes/lipid complexes (Doxil^®^, Onivyde^®^, and Vyxeos^®^) [16,17,18]; albumin nanoparticles (Abraxane^®^) [19]; and polymeric nanoparticles (Genexol^®^-PM) [20], have achieved clinical success in enhancing intratumoral delivery of chemotherapeutics, improving antitumor efficacy while reducing systemic side effects. Building on this success, incorporating antibacterial agents into nanocarriers offers a targeted strategy to eradicate intratumoral bacteria, potentially modulating the TME and enhancing cancer treatment responsiveness [21,22]. In addition, developing precision nano-based strategies that selectively target pathogenic species within particular tumor types while minimizing disruption of commensal communities may prove to be due to the bacteria-specific contributions in different tumors [4].

Given the early stage of this emerging field, this scoping review aims to provide a comprehensive summary of current research on various nanotechnologies designed to target intratumoral bacteria. Special focuses are placed on (i) the types of cancers closely correlated with intratumoral bacteria; (ii) animal models and (iii) bacterial species adopted to establish infected tumor models; (iv) nanodrug-based strategies and (v) their therapeutic outcome evaluation approaches. In this context, this review evaluates experimental approaches used to establish intratumoral infection models, assesses in vivo efficacy of antibacterial nanodrugs, and explores their impact on TME immunity. On the basis of the collected knowledge, we then outline the prospects and challenges of nano-therapies in clinical application. Ultimately, this review seeks to guide future research directions and facilitate the translation of promising nanomedicines for effective management of bacteria-infected tumors.

## 2. Materials and Methods

A scoping review approach was employed to address the wide-ranging diversity of bacterial species, cancer types, and nanotechnologies explored in studies targeting infected tumors. Using the scoping review framework, we aimed to delineate critical gaps in the current literature and identify key advancements. This approach can facilitate the characterization of existing evidence on therapeutic effectiveness and provide a foundational understanding of the novel nanodrug strategies across oncological and microbiological contexts, providing useful information on future systematic reviews and guiding translational research efforts in this rapidly evolving field.

The present scoping review has followed PRISMA-ScR (2018) guidelines, and the detailed protocol has been registered on Open Science Framework (OSF).

### 2.1. Search Strategy

All reference publications included in this review were retrieved on 1 August 2025 from three major databases—Web of Science, PubMed, and Scopus. The search keywords were “(kill* OR eradicate* OR eliminate*) AND intratumoral AND (bacteria OR infection)”. The inclusion criteria were restricted to research articles, with no limitations on publication date. Duplicate records were removed using EndNote 20 (Clarivate, London, UK) and manual screening.

### 2.2. Eligibility Criteria

Clinical studies, case reports, reviews, and studies adopting probiotics or bacteria as an antitumor approach were excluded in this study. Studies chosen in this review were based on the following criteria: involvement of tumor-associated pathogenic bacteria, in vitro or in vivo antimicrobial studies, and in vivo anticancer efficacy in bacteria-infected tumor-bearing animal models (Table 1).

### 2.3. Data Extraction

Data from eligible studies were extracted and listed. Key information collected included author names, publication years, investigated bacterial strains, cancer types, animal models used, inoculation methods, nano-based therapeutic strategies developed to kill intratumoral bacteria, and the underlying mechanisms of actions.

## 3. Results

**Figure 1:**
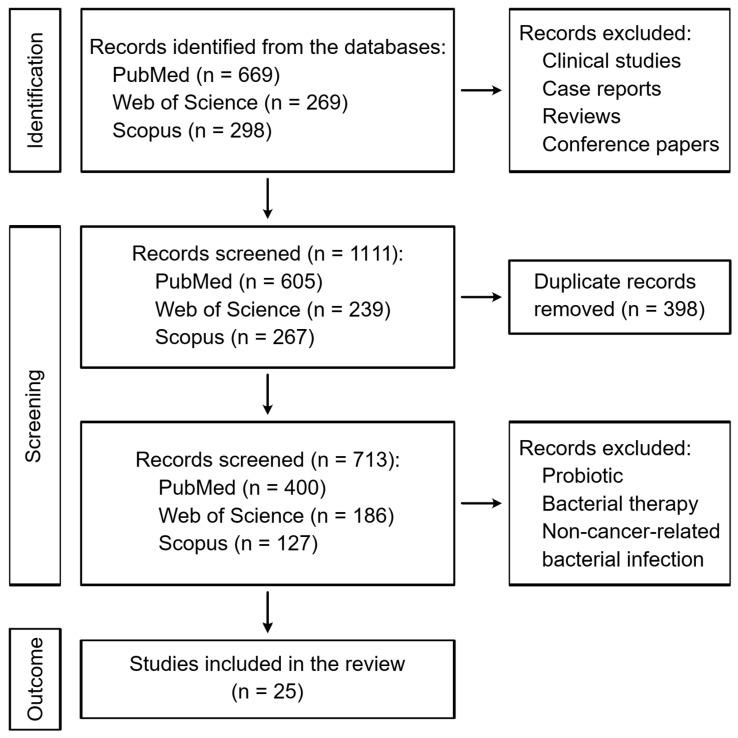
Flow diagram of the literature selection.

A total of 1236 articles were initially identified across the three selected databases. After excluding non-research articles, 1111 studies remained. Following the removal of duplicate articles, a collection of 713 articles was retained for further screening. Subsequent evaluation led to the exclusion of publications with irrelevant topics, resulting in 25 research articles being included in this scoping review. The selection process is illustrated in Figure 1. Key findings of the included studies are listed in Table 2.

### 3.1. Cancer Types

While the majority of selected studies focused on a single cancer type, five publications evaluated their proposed nano-based technologies across multiple cancer models [23,27,29,33,38]. Of the twenty-five included studies, nineteen investigated CRC, using human, murine, or both cancer models (Table 1). Other cancer types investigated included breast cancer [23,24,27,34,38], pancreatic cancer [37,39], cervical cancer [27], melanoma [33], lung cancer [29,35], and liver cancer [23].

### 3.2. Animal Models

All included studies validated the antibacterial and/or antitumor efficacy of the designed nanosystems against at least one selected bacterial strain in murine cancer models. BALB/c mice were the most frequently used species, while other strains such as C57BL/6, NU/J, and genetically engineered C57BL/6J-*Apc*^Min/+^ mice were employed (Table 1). In addition, human tumor cell xenograft models were adopted in six studies using BALB/c nude mice.

Due to the ease of model construction and convenient tumor size monitoring, subcutaneous (s.c.) xenograft models were adopted in twenty-two studies. After initial confirmation of in vivo efficacy in s.c. models, nine studies further evaluated therapeutic effects in orthotopic cancer models, which are reconsidered more clinically relevant because they can better recapitulate the TME and metastatic behavior [46]. To monitor tumor growth in these models, luciferase (Luc)-expressing cancer cells are often used to enable in vivo bioluminescence imaging [47]. Among the studies utilizing orthotopic models, one reported lung cancer metastasis [34]. Since metastasis is a hallmark of cancer [48], one study specifically established a lung metastasis model by intravenously injecting HCT116 cells into BALB/c nude mice to evaluate the efficacy of nanodrugs in mitigating bacteria-induced metastasis [45]. Liver metastasis was observed in a separate study, where murine CRC cells were implanted by hemi-splenic injection [14]. Spontaneous tumor models were also used in five studies to mimic human disease progression more closely. These included chemically induced CRC models using azoxymethane/dextran sodium sulfate [10,28,33,35] and genetically modified models such as *Apc*^Min/+^ mice [41].

### 3.3. Bacterial Strains and Inoculation

*F. nucleatum*, a Gram-negative anaerobic oral commensal bacterium, is frequently detected in CRC tissues and associated with poor prognosis and chemoresistance [49]. Mechanism studies show it induces protective autophagy through TLR4–MyD88 signaling and modulates epithelial–mesenchymal transition (EMT) markers in a p53-dependent manner [50]. Thirteen studies examined *F. nucleatum*-associated CRC using *s.c.* tumor models, with infections mostly established by direct intratumoral injection or, less commonly, tail vein injection [25,30,32]. Pre-infected cancer cells were also used to investigated liver [14] and lung [45] metastasis. To better simulate clinical conditions, oral gavage was employed in eight studies with orthotopic [14,32,36,42,45] and spontaneous [28,33,41] tumor models.

Other oral microbiome members, including *Peptostreptococcus anaerobius* and *B. fragilis*, have also been reported to exacerbate CRC development, enhance chemoresistance, and modulate tumor immunity [51,52]. They promote oncogenesis through mechanisms, such as myeloid-derived suppressor cells (MDSCs) recruitment, EMT activation, induction of cancer stemness, and modulation of host signaling pathways like NOTCH1 and β-catenin [4]. Their enrichment in CRC tumor tissues highlights their potential as therapeutic targets. Three studies investigated these bacteria, establishing intratumoral infections via direct injection or oral gavage in subcutaneous and orthotopic CRC models [10,29,36].

*E. coli*, particularly polyketide synthase-positive (pks^+^) strains producing the genotoxin colibactin, are increasingly detected in tumor tissues and linked to DNA damage, genomic instability, and cancer development [53]. Five studies investigated intratumoral *E. coli* across breast [24], CRC [23], pancreatic [37,39], and lung [35] cancers. Infections were typically induced via intravenous [24,39] or direct intratumoral [23,35] injections, while one pancreatic cancer model used pre-infected Panc02 cells to establish the s.c. tumor model [37].

*Staphylococcus aureus*, a Gram-positive opportunistic pathogen capable of colonizing various anatomical sites and causing infections ranging from self-limiting to life-threatening in humans [54], was also studied in three cancer models, including breast [27], CRC [29], and lung [35]. In a post-surgical infection management study, *S. aureus* was subcutaneously injected into surgical sites where 99% of the subcutaneously implanted tumor tissues had been removed, simulating residual tumor bed infection [27]. In the other two studies, direct intratumoral injection was used [29,35], highlighting the reliability of this method in achieving localized colonization and consistent infection dynamics.

Tumors are increasingly recognized as habitats for diverse bacteria communities [4]. To better simulate this polymicrobial environment, two studies introduced bacterial mixtures directly into tumor sites, such as combinations of *E. coli*, *S. aureus*, *Staphylococcus intermedius*, and *Prevotella intermedia* [35] or *F. nucleatum* and other commensal bacteria [34]. In one orthotopic CRC mouse model, *Peptostreptococcus anaerobius* and *F. nucleatum* were co-administered through oral gavage to reflect microbial heterogeneity [36]. Another unique study transplanted fecal microbiota from untreated to drug-treated tumor-bearing mice, revealing the influence of microbiota on therapeutic outcomes and tumor recurrence [38].

Although various approaches were employed to establish in vivo intratumoral infections across different cancer models, all studies confirmed the co-localization of bacteria and tumor cells in the same vicinity. Tumor-resident bacteria exhibited varying degrees of influence on tumor growth, distant dissemination, and therapeutic responses by altering signaling pathways or inactivating drugs through bacteria secretions in the TME [55]. Emerging nano-based therapeutics targeting these bacteria represent promising strategies for cancer management.

### 3.4. Nano-Based Drug Treatment Strategies

**Figure 2:**
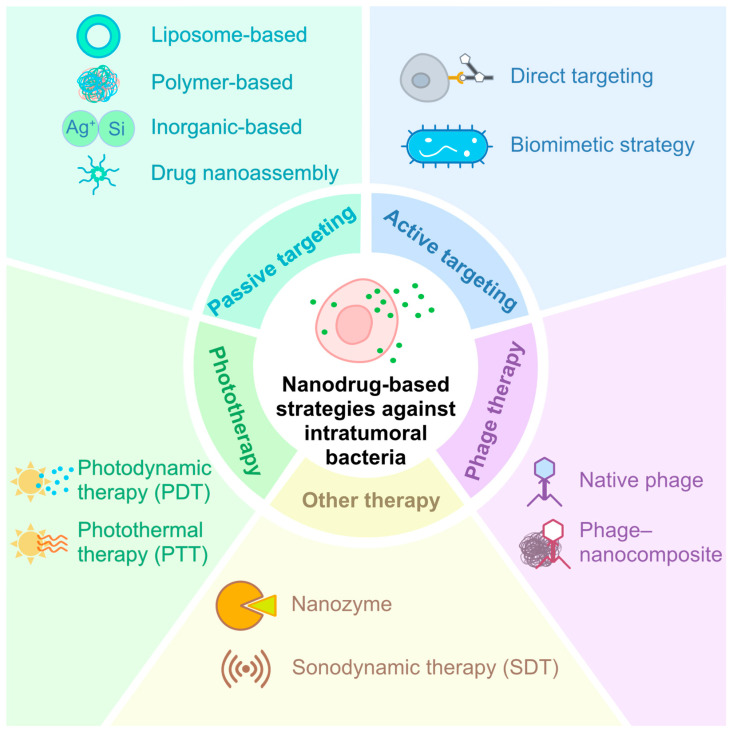
Classification of the nanodrugs targeting tumor-associated bacteria.

Recent advances in nanotechnology have opened new avenues for targeting intratumoral bacteria, offering innovative strategies to overcome bacteria-induced tumor progression and therapeutic resistance. Various nano-based drug delivery systems have been designed to simultaneously inhibit bacterial proliferation and suppress tumor growth [56]. These systems can be broadly classified into five categories: passive targeting nanodrugs, active targeting nanodrugs, phototherapy, phage therapy, and other emerging modalities (Figure 2).

Basically, nano-based therapeutic approaches mainly target the TME by either physiochemical property (e.g., size, charge, pH, or GSH) or specific binding to certain pathogenic bacteria or tumors based on ligand–receptor interactions. For the latter scenario, some representative examples of specific binding targets are expounded (Table 3). Among active targeting strategies, corresponding ligands such as small molecules or antibodies targeting specific bacteria or tumors were anchored to the synthesized nanodrugs. Moreover, extracted bacterial membranes or inactivated bacteria were employed in three studies adopting a biomimicking strategy to modify the nanoparticles owing to their inherent enrichment in CRC. As for phage therapy, the specificity of phage to host bacteria was utilized in both native phage therapy and phage–nanocomposites (Table 3).

#### 3.4.1. Passive Targeting Drug Delivery

Passive targeting nanodrugs for cancer treatments mainly harness the EPR effects of tumors, where leaky vasculature and impaired lymphatic drainage allow nanoparticles to accumulate at tumor sites [57,58]. Successful design requires particles within the 10–200 nm range, PEGylation to increase hydrophilicity, and a neutral to slightly negative surface charge to prolong circulation and reduce clearance [57,58]. To date, all US Food and Drug Administration (FDA)-approved cancer nanomedicines rely on passive targeting, suggesting its high clinical relevance and translation potential [59]. Therefore, this approach has also been proposed to target intratumoral bacteria.

- Liposome-Based Nanosystems

Liposomes are among the most widely accepted nanocarriers in drug delivery due to their excellent biosafety profile, versatile drug-loading capacity, and effective passive targeting ability [60]. Encapsulating antibacterial agents into liposomes to target intratumoral bacteria has been attempted. Wang et al. developed tinidazole–silver complex liposomes (LipoAgTNZ), which readily dissociate in acid environments to kill *F. nucleatum* and *E. coli* residing within CRC [14]. Bacterial lysis released cancer-specific microbial neoantigens, priming T cells to recognize both infected and uninfected tumors, resulting in significant tumor reduction [14]. Differently, Busscher and co-workers co-encapsulated an anticancer drug (gemcitabine) and an antibiotic (ciprofloxacin) into pH-responsive liposomes (DCPA-H_2_O), which protonated and expanded under an acidic environment to release both drugs at similar kinetics [23]. This dual-delivery strategy simultaneously eradicated *E. coli* and cancer cells, overcoming chemoresistance caused by bacterial deactivation of gemcitabine. Both liposomal systems employed PEGylation to prolong circulation and enhance tumor accumulation, achieving reductions in bacterial burden and tumor volume while minimizing systemic antibiotic side effects and preserving normal microbiota. By harnessing the acidic TME to trigger drug release, these liposomal nanosystems further reduced off-target toxicity.

- Polymer-Based Nanosystems

Natural and synthetic biodegradable polymers hold great promise for nanoparticle-based drug delivery systems and have been extensively studied for cancer treatments [61]. Grafting antibiotics and chemotherapeutics onto polymer chains for subsequent micelle/nanoparticle formations represents an effective strategy, allowing precise control of the drug ratio [62]. Using the grafting technique, Qiu et al. designed a Pluronic F127-based micelle system co-delivering gemcitabine and colistin to selectively target intratumoral *E. coli* and protect induced drug resistance and validate the efficacy in a murine breast cancer model [24]. Similarly, Li et al. developed a multifunctional amphiphilic polymer system (OPPL) impregnated with lauric acid (as antibacterial agent), a platinum prodrug, and oleic acid-modified superparamagnetic iron oxide nanoparticles (O-SPIONs@PG-Pt-LA, OPPL) [25]. In a *F. nucleatum*-associated CRC murine model, the OPPL-based nanodrug demonstrated improved drug accumulation at the tumor site, effectively suppressing tumor growth and inhibiting bacteria growth. Mechanism investigation revealed that the O-SPION component could exert distinctive peroxidase-like activity to induce tumor ferroptosis and synergize with the incorporated chemotherapeutic and antibiotic in killing the cancer and bacterial cells. These works highlight the therapeutic potential of polymer-based dual-delivery nanosystems for infected tumors, though challenges such as immunogenicity, synthesis scalability, and optimization of drug release kinetics in the heterogeneous TME remain barriers to clinical translation.

- Inorganic Nanosystems

Inorganic nanoconstructs are emerging as powerful theragnostic tools in cancer treatment, with growing interest in their application against infected tumors. Among these, dendritic mesoporous silica nanoparticles (DMSNs) are particularly attractive because their ultra-large porous structures can accommodate diverse functional and therapeutic agents [63]. Chen et al. engineered DMSNs via a coordination–redox strategy to achieve homogeneous in situ growth of ultrasmall antibacterial silver nanoparticles within the mesopores, which were subsequently loaded with a chemotherapeutic drug, epirubicin (EPI), to generate antibacterial–antineoplastic Ag@DMSNs-EPI nanoparticles (ADENs) [26]. This system effectively eliminated *F. nucleatum* in CRC by blocking bacteria-induced autophagy, overcoming chemoresistance and enhancing therapeutic efficacy. Beyond systemic delivery, Chu et al. explored local treatments with an in situ fibrin gel loaded with fluorescent flavonoid–silica nanocomposites (FSiNCs@Fibrin). The nanocomposites could selectively accumulate ROS in both tumor and bacterial cells to trigger cell death. When incorporated with fibrinogen and thrombin, the gel solution could be directly sprayed onto surgical sites for post-operative treatment, achieving an 18-fold reduction in intratumoral bacteria and a 12-fold decrease in tumor regrowth compared to the free flavonoid-loading gel [27]. This pioneering work opens new therapeutic strategies for post-surgical cancer management, potentially minimizing tumor recurrence following resection, particularly those driven by bacteria.

- Drug Nanoassemblies

The use of excipients in nano-formulations may pose safety concerns [64], prompting interest in pure drug self-assembled nanosystems in cancer therapy. These carrier-free platforms offer distinct advantages, including high drug loading, simplified preparation, and the ability to co-deliver multiple agents for combination therapy [65]. Gao et al. developed an innovative approach by linking the antibiotic metronidazole (MTI) with the chemotherapeutic agent fluorouracil (FDU) by a 3,3′-dithiodipropionic acid with disulfide bonds to create an amphiphilic molecule. This conjugate spontaneously self-assembled into nanoparticles (MTI–FDU) in aqueous solution, requiring no additional excipients. The disulfide bond was responsive to elevated glutathione levels in the CRC TME, enabling controlled release of both drugs. In two CRC models (AOM/DSS-induced and CT26 *s.c.* mouse models) with *F. nucleatum* infiltration, MTI–FDU nanoassemblies achieved dual targeting of intratumoral bacteria and tumor cells with minimum disruption to the gut microbiome homeostasis [28]. This innovative nanodrug formulation warrants further development to assess its translational potential in managing infected tumors.

#### 3.4.2. Active Targeting Drug Delivery Systems

Nanoparticle systems can achieve active targeting by decorating their surface with targeting moieties that enhance therapeutic specificity and, hence, efficacy. Although several actively targeted cancer nanomedicine candidates are under clinical trials, none have yet received approval from the FDA or European Medicines Agency (EMA) [66]. In the context of bacteria-associated cancer, active targeting strategies generally fall into two categories: (i) direct targeting, which employs chemical or biological ligands to recognize specific bacterial or cancer cell components, and (ii) biomimetic targeting, which mimics bacterial surface properties to facilitate tumor recognition.

- Direct Bacterial Targeting and Cancer Cell Targeting

Modifying nanosystems with targeting ligands that recognize bacterial or tumor associated markers offer a promising approach to selectively target intratumoral bacteria [67,68]. Song et al. designed mesoporous silica nanoparticles (MSNs) functionalized with lipoteichoic acid (LTA) antibody, enabling specific binding to Gram-positive bacteria and localized drug release within the TME [29]. This strategy augmented drug accumulation by approximately three-fold in colorectal, lung, and breast cancer models and demonstrated effective tumor suppression along with improved survival rates in a CRC model. In another study, Li et al. employed phenylboric acid (PBA), which binds sialic acid epitopes overexpressed on cancer cells, to functionalize a dual-drug nanoassembly system to target *F. nucleatum*-associated CRC [30]. In this design, lauric acid (LA) and PBA-conjugated oligomethyleneimine (OEI) were linked with platinum(IV) prodrug-modified polyglycidyl ether (PG) via pH-responsive boronate ester bonds to produce OLP/PP nanoassemblies. Controlled drug release was enabled in the acidic TME to effectively eliminate both extracellular and intracellular *F. nucleatum*, overcoming the bacteria-induced chemoresistance. Sequential drug release may further enhance efficacy, with the initial antibacterial release clearing intratumoral bacteria and reducing drug metabolism, followed by sustained release of anticancer drugs to eliminate cancer cells [69]. Building on this concept, Xie et al. developed size-tunable nanogels featured with cascaded dual-drug release profiles [31]. Metronidazole was rapidly released upon hyaluronidase-triggered swelling to eliminate *F. nucleatum* in the tumor tissues, followed by sustained release of doxorubicin incorporated in the folate-decorated zinc-imidazolate frameworks for targeted cancer therapy. This system achieved 85.7% tumor growth inhibition and doubled the median survival in CRC mouse models. Collectively, these studies demonstrated the potential of ligand-functionalized nanosystems to enhance bacterial and cancer cell targeting.

- Bacterial Membrane-Mimicking Strategies

As specific bacterial species are enriched in tumors, mimicking bacterial surfaces has emerged as an innovative strategy to achieve tumor targeting [70]. Chen et al. exploited the high affinity between *F. nucleatum* membrane protein Fap-2 and Gal-GalNAc overexpressed on CRC cells to design Colistin-LipoFM, a liposomal system fused with extracted *F. nucleatum* cytoplasmic membranes. This biomimetic nanomedicine achieved 1.5-fold higher tumor accumulation, selectively eliminated tumor-colonized *F. nucleatum*, and restored the efficacy of anti-CTLA4 and anti-PD-1 immunotherapy by reducing MDSCs and reactivating CD8^+^ T cells in a CRC murine model [33]. The LipoFM platform was later extended to breast cancer, where *F. nucleatum* colonization was detected in 62.5% of clinical samples. The colistin-LipoFM restored chemotherapy sensitivity in *F. nucleatum*-infected breast tumors, while doxycycline-LipoFM showed broader antimicrobial activity; suppressed bacteria-induced lung metastasis; and significantly enhanced efficacy of standard cyclophosphamide (CTX), methotrexate (MTX), and 5-FU (CMF) chemotherapy, achieving complete tumor regression in mice [34].

In lung cancer, Han et al. uncovered that *E. coli* and *S. aureus* residing in the lungs could deactivate chemotherapy drugs, such as etoposide and gemcitabine, forming less-effective metabolites [35]. To counter this, they designed inhalable gallium-polyphenol nanoparticles decorated with capsular polysaccharide extracted from *Streptococcus pneumoniae* (GaTa-CP), providing immune stealth properties to enhanced lung retention. Released Ga^3+^ ions acted as iron mimetics, deceiving iron uptake systems to disrupt bacterial iron metabolism. This mechanism effectively killed intratumoral bacteria and prevented drug degradation. In orthotopic lung cancer models, GaTa-CP@etoposide achieved ~85% tumor inhibition versus ~15% with free etoposide, mainly driven by effective microbiota elimination and a 3.1-fold increase in intratumoral drug concentration.

Zhuang et al. took a different approach by developing a biomimetic nanovaccine (PMO) using inactivated *P. anaerobius*, a CRC-enriched bacterium that expresses PCWBR2 and specifically binds to α2β1 integrin on CRC cells, as a carrier for MnO_2_ and oxaliplatin [36]. Under the acidic tumor conditions, MnO_2_ dissociated with Mn^2+^ and catalyzed endogenous H_2_O_2_ to generate hydroxyl radicals, killing both tumor cells and tumor-associated bacteria. PMO treatment also remodeled the tumor microbiome by suppressing pro-tumoral bacteria and restoring beneficial microbiota, reversing the immunosuppressive TME. Specifically, it decreased the proportion of MDSCs and M2-type macrophages, both of which are known to inhibit antitumor immunity and promote immune evasion. Concurrently, T-cell infiltration, particularly cytotoxic CD8^+^ T cells, was promoted. These collectively reactivated immune surveillance and amplified the therapeutic response that near-complete tumor suppression was noted in the CRC mouse model.

Together, these studies highlight biomimetic nanomedicines as powerful tools for synergistic elimination of intratumoral bacteria and cancer cells. The multifunctional platforms can simultaneously deliver antibiotics, chemotherapeutics, and immune modulators, enabling enhanced tumor specificity, restoration of drug sensitivity, and reprogramming of the TME. However, the complexity of manufacturing biomimetic systems, especially those incorporating bacterial components, poses significant challenges in scalability, reproducibility, and regulatory approval.

#### 3.4.3. Systems Enabling Phototherapy

Phototherapy, encompassing photodynamic therapy (PDT) and photothermal therapy (PTT), has emerged as a promising approach for eradicating intratumoral bacteria. These light-triggered therapies offer high spatial precision, minimal invasiveness, low drug resistance, and the potential for synergistic integration with nanotechnology [71].

PDT relies on photosensitizers that generate ROS under light irradiation to selectively kill cancer cells or bacteria [71]. Conventional photosensitizers, such as the chlorophyll-derived pyropheophorbide-a (Ppa), often suffer from aggregation-induced quenching (ACQ), which limits their fluorescence and therapeutic efficacy in the aggregated state [72]. To address this, Liu et al. conjugated Ppa with an antimicrobial peptide, ubiquicidin (UBI) 29–41, via a PEG linker, forming amphiphilic micelles (UPPMs) to increase its stability in aqueous solution [37]. Under light irradiation, UPPMs generated ROS to effectively kill *E. coli* and *S. aureus* while also enabling fluorescence imaging of bacterial localization, highlighting its dual therapeutic and diagnostic potential. More broadly, the discovery of aggregation-induced emission (AIE) has enabled the development of photosensitizers that maintain strong fluorescence and therapeutic efficacy in the aggregated state [72,73,74].

PTT, in contrast, utilizes photothermal agents that convert near-infrared (NIR) light into localized heat to ablate bacteria and tumor cells [71]. Kong et al. designed an inorganic nanoplatform (Nb_2_C/Au/anti-TNF-α-PVP) in which antibacterial gold nanoparticles were anchored onto Nb_2_C nanosheets and conjugated with an anti-TNF-α drug to suppress inflammation [38]. Polyvinyl pyrrolidone (PVP) was also included to stabilize the nanocomposite and prevent drug leakage. In another study, Kang et al. synthesized an amphiphilic glutathione (GSH)-responsive polymer (SGP) with potent antibacterial activity [39]. Gemcitabine (Gem) and a photothermal agent, IR1048, were encapsulated in polymeric nanoparticles, which were subsequently coated with a hyaluronic acid (HA) shell to form dual-cascade GSH-responsive nanoparticles (sNP@G/IR). Upon NIR irradiation, sNP@G/IR exhibited efficient bacterial eradication through photothermal effects and drug release, demonstrating synergistic antibacterial efficacy.

Despite the promising results of PDT and PTT in preclinical models, several challenges remain for clinical translation. These include limited tissue penetration of light sources, potential off-target phototoxicity, variability in bacterial colonization across tumor types, and the complexity of designing biocompatible, scalable nanomaterials with consistent performance in human systems. Moreover, regulatory hurdles and the need for precise control over light delivery in deep-seated tumors further complicate their adoption in routine oncology practice. Addressing these barriers will be essential to fully realize the therapeutic potential of PDT and PTT in combating intratumoral infections and enhancing cancer treatment outcomes.

#### 3.4.4. Phage Therapy

Humans are colonized not only by bacteria but also phages, which outnumber bacteria by a factor of 10 and represent the most abundant foreign microorganisms in the human body [75,76]. Far from being passive residents, phages play crucial roles in maintaining human health. For example, intestinal phages act as protective barriers to prevent colonization of pathogenic bacteria and help to maintain microbial balance [77]. With the ever-escalating antibiotic resistance crisis, phages have been revitalized to treat infections associated with antibiotic-resistant “superbugs” [78]. Their excellent host specificity makes them ideal for precision targeting of intratumoral bacteria. However, the physiological stresses and limited tissue penetration can hinder effective phage delivery [79]. To address these challenges, phage–nanocomposite systems have been developed to enhance the functionality of phages.

- Phage Systems for Bacterial Targeting

Phage therapy has been investigated to treat CRC infections without disturbing the surrounding commensal microflora [80]. Targeted phage therapy can selectively eliminate resistance-inducing intratumoral bacteria, such as *B. fragilis* and *F. nucleatum*, restoring chemotherapy sensitivity and promoting immune remodeling within the TME [10,40]. Specifically, Ding et al. demonstrated that lytic phage VA7 effectively disrupted *B. fragilis*-tumor interactions and restored the efficacy of 5-FU and OXA in both AOM/DSS-induced CRC and *Apc*^Min/+^ CRC mouse models [10]. Similarly, Lam et al. reported that targeting *F. nucleatum* with phage ØTCUFN3 could significantly reduce the bacterial abundance in tumor tissues, facilitating the modulation of EMT markers in a p53-dependent manner [40]. In vivo experiments also confirmed that phage treatment could reduce tumor volume and weight, but the underlying p53-dependent mechanisms remain to be investigated.

Although native phages offer high specificity, their narrow host range limits clinical utility [81]. Engineered phages can overcome this constraint by expanding host targeting via tail fiber modification and programing CRISPR–Cas systems that target essential and virulent genes across diverse bacterial strains. For example, the SNIPR biome developed SNIPR001, a phage cocktail containing four tail fiber-engineered phages armed with CRISPR–Cas systems targeting *E. coli* genes (lptA, murA, bolA, rpoH, and fimH). SNIPR001 covered 90.4% of 382 clinical isolates of *E. coli* and achieved a 4-log bacterial reduction in mice [82]. It has recently been granted with Fast Track Designation by the FDA and is in clinical trials for preventing *E. coli* bacteremia in hematological cancer patients at risk of neutropenia [82]. This marks a milestone of phage therapy in precision microbiome modulation for cancer-related infection management.

- Phage–Nanocomposite Systems for Enhanced Delivery

Phage–nanocomposite systems combine the natural specificity of phages with the versatility of nanocarriers to enhance the therapeutic potential of phage-based treatments. These platforms extend the function of phages beyond antibacterial activity, enabling them to act as precision-guided delivery vehicles and immune modulators. The nanocarriers also protect phages under harsh conditions, such as gastric acidity [79]. For example, Zhao et al. developed a DNA nanosheet–phage system (DNPs@P) to improve oral delivery of phages for *Streptococcus gallolyticus* targeting [43]. The DNA origami structures shielded phages from reactive oxygen species and acidic conditions, improving viability by 1.85-fold and ~8800-fold, respectively. In AOM+DSS+*Sg*-induced colitis-associated cancer models, the DNPs@P completely prevented tumor formation, demonstrating the importance of eliminating *S. gallolyticus* to block inflammation-to-cancer transition [43]. In CRC treatments, Zheng et al. designed a phage-guided nanosystem using dibenzocyclooctyne (DBCO) click chemistry to conjugate P2 phages with irinotecan-loaded dextran nanoparticles [29]. This nanosystem not only restored drug sensitivity by suppressing *F. nucleatum*-induced autophagy but also promoted *Clostridium butyricum* proliferation and increased colonic short-chain fatty acid production to regenerate a healthier gut microenvironment. This strategy yielded a three-fold increase in intratumoral drug accumulation, reduced adenoma numbers from over 20.0 to 8.2 and extended the median survival from 20 to 42 days in *Apc*^Min/+^ mouse models. Separately, Dong et al. engineered a M13@Ag hybrid system, where silver (Ag) nanoparticles were electrostatically assembled on the surface of M13 phages specifically binding to *F. nucleatum* [42]. Ag nanoparticles in this dual-action system selectively cleared *F. nucleatum* in the TME and blocked the recruitment of immunosuppressive cells, while M13 phages stimulated the host immune responses with their native protein coat to boost DC maturation and macrophage polarization. Combined treatment with immune checkpoint inhibitor α-PD-1 and chemotherapeutics significantly delayed orthotopic CRC tumorigenesis, as well as prolonged the survival time of mice. Such synergistic effects highlight the important potential of selectively modulating the gut microbiota to neutralize the immunosuppressive TME using phage–nanocomposite systems in CRC treatment.

#### 3.4.5. Other Nano-Based Therapies

Nanozyme therapies represent innovative nanomaterial-based strategies to overcome *F. nucleatum*-mediated resistance in CRC [44]. Wang et al. developed a bovine serum albumin-supported copper single-atom nanozyme (BSA-Cu SAN) with a biomimetic CuN_2_O_2_ coordination structure. This nanozyme system demonstrated effective peroxidase-like activity, converting H_2_O_2_ into cytotoxic ·OH radicals while depleting glutathione, inducing oxidative stress that killed *F. nucleatum* through bacterial membrane disruption. By disrupting TLR4–MyD88 signaling and miRNA-mediated autophagy, BSA-Cu SAN restored normal autophagy levels and sensitized cancer cells to oxidative damage, achieving up to 82.97% tumor growth inhibition. Its protein-based design also enabled rapid renal clearance (t_1/2_ = 1.55 h), alleviating concerns about heavy metal accumulation [44].

Sonodynamic therapy (SDT) provides an alternative activation method using ultrasound, addressing limitations of light penetration and phototoxicity [83]. Qu et al. developed Au@BSA-CuPpIX nanocomposites, embedding gold nanoparticles in BSA and modifying them with the antibacterial sonosensitizer CuPpIX [45]. This platform targeted *F. nucleatum*-mediated resistance and porphyrin-associated skin phototoxicity. Under ultrasonic stimulation, the nanocomposites generated singlet oxygen to eliminate *F. nucleatum* and induce tumor cell apoptosis. Gold nanoparticles provided selective photoprotection, reducing light-induced ROS by 76% while preserving ultrasound-triggered ROS generation. In xenograft and orthotopic colorectal cancer models, the treatment achieved 94% tumor inhibition and extended survival while also reducing lung metastasis and preventing skin photodamage.

Together, these approaches highlight the versatility of nanomedicine in modulating tumor–bacteria interactions, offering multifunctional platforms to enhance therapeutic outcomes in CRC.

## 4. Discussion

Recent studies have revealed that certain bacterial species are significantly more abundant within tumor tissues compared to adjacent normal tissues. Emerging evidence also reveals that intratumoral bacteria indeed play critical roles in promoting cancer progression and contributing to resistance against conventional cancer therapies. These bacteria contribute to tumor progression mainly through supporting tumor proliferation, inactivating chemotherapeutic agents and modulating immune responses to favor tumor survival. The underlying mechanisms linking intratumoral bacteria to tumor progression have been comprehensively reviewed in the recent literature [4,5]. Building on these insights, targeting intratumoral bacteria has emerged as a multifaceted therapeutic strategy that integrates antimicrobial, chemotherapeutic, and immunomodulatory effects to mitigate tumor-associated infections and overcome drug resistance [84,85]. In particular, nano-based delivery systems are being explored as precision tools to selectively eliminate tumor-resident bacteria while simultaneously enhancing drug efficacy, with acceptable safety profiles.

### 4.1. Efficacy Evaluation

#### 4.1.1. Antibacterial Effect Evaluation

In vivo antibacterial efficacy of nanosystems was typically assessed after in vitro optimization. The most common method was plate counting of intratumoral colony-forming units (CFUs) from excised tumor samples. Additional techniques included fluorescence in situ hybridization (FISH) [14,26,28,35,41,42,44,45]; Giemsa staining [31]; and immunohistochemistry (IHC) for lipopolysaccharide (LPS) [28], RT–qPCR [26,40], and 16S rDNA/rRNA sequencing [10,32,41,42]. While these methods required post-mortem analysis, bioluminescence imaging, using genetically engineered bioluminescent bacteria, enabled real-time, non-invasive monitoring of dynamic changes in bacterial populations in infectious tumor-bearing mouse models, reducing the need for animal sacrifice [23,37]. Across all methods, nanosystems consistently achieved significant bacterial reduction in vivo.

A key therapeutic goal of antibacterial nanosystems is to selectively eliminate harmful intratumoral bacteria while preserving normal microbiota. Gut [10,14,28,33,36,37,41,43], fecal [42] and lung [35] microbiota analyses demonstrated that nanosystems such as UPPM nanomicelles [37], silica nanoparticles [26,27,29], hybrid phage [41,42,43] and biomimetic nanovaccine [36] could reduce tumor-promoting bacteria without disrupting microbial homeostasis. Some approaches even restored microbial diversity, including enrichment of tumor-suppressive strains like *C. butyricum* [41]. These findings highlight the promise of nanosystems that precisely target harmful bacteria while safeguarding beneficial microbiota, minimizing off-target effects and supporting overall health.

#### 4.1.2. Antitumor Effect Evaluation

Some nanosystems are designed solely to target the intratumoral bacteria without directly killing cancer cells, such as the silver–tinidazole liposomes (LipoAgTNZ) designed by Wang et al., but they reversed bacteria-induced upregulation of migration and DNA damage-related markers [14]. Other nanosystems have combined anticancer and antibacterial agents for synergistic effects. For instance, nanogels with doxorubicin and metronidazole enhanced cytotoxicity against CRC compared with single agents [31], while a PMO formulation containing metronidazole, oxaliplatin, and MnO_2_ reduced cell viability by ~60% versus ~20% with individual components [36]. These findings show that nanodrugs can exert anticancer activity both through direct cytotoxicity and by modulating cancer cell signaling pathways.

In vivo studies of nanodrugs in bacteria-infected tumors consistently demonstrated strong tumor suppression. Subcutaneous models showed reduced tumor size through volume and weight measurements, while orthotopic luciferase-expressing models enabled real-time bioluminescence monitoring and revealed near-complete eradication in some cases [14,29,32,34,35,36,41,42,45]. Spontaneous tumor models, which preserve the native TME, provide clinically relevant insights into cancer progression [86,87]. In six studies, nanodrug treatments reduced tumor nodules or even realized total regression through observing the excised tissues or ^18^F-fluorodeoxyglucose positron emission tomography/computed tomography (^18^F-FDG-PET/CT) imaging, indicating strong antitumor efficacy [10,28,33,35,41,43]. Metastasis, a major hallmark of cancer progression [48], was also effectively suppressed in specialized models [14,34,45], confirming the potent antimetastatic activity of several nanosystems.

Intratumoral bacteria are increasingly recognized for their role in promoting chemoresistance, compromising the efficacy of current anticancer therapies [49,53,88,89]. Eleven studies demonstrated that nanodrugs could overcome bacteria-induced resistance [10,24,26,30,33,34,35,37,39,41,42], with five specifically targeting *F. nucleatum* in CRC [26,30,33,34,41]. By clearing the bacterial burden within tumors, the studied nanosystems effectively restored tumor sensitivity to conventional treatments like 5-FU [10,34], OXA [10,30] or immunotherapy [33,42].

#### 4.1.3. Immunity Regulation

Intratumoral bacterial infections may contribute to cancer progression by inducing inflammation and shaping an immunosuppressive TME [90,91,92]. For example, the pro-cancer bacterium *B. fragilis* has recently been reported to increase immunosuppressive regulatory T cells (Treg) in CRC, thereby promoting an immune-suppressive environment [10]. Another case was *F. nucleatum* inhibiting natural killer (NK) cells and CD4^+^ T cells [93]. To counter these effects, nanosystems have been developed that both suppress bacteria-induced inflammation and enhance antitumor immunity. Nanoplatforms like PG-Pt-LA/CB reduced inflammatory cytokines IL-6 and TNF-α [32], while Au@BSA-CuPpIX downregulated TLR4, MyD88, and p65 by eliminating *F. nucleatum* [45]. Apart from dampening inflammatory, nanosystems can also reprogram immune responses by enhancing CD8^+^ cytotoxic T-cell infiltration [14,28,33,36,38,39,42], reducing immunosuppressive cells such as MDSCs and Tregs [28,33,36,42], and selectively targeting protumor macrophages while preserving antitumor subsets [14,36,42]. Examples include the PMO nanovaccine, which decreased M2-type tumor-associated macrophage (TAM)-linked resistance [36], and the phage-based M13@Ag system, which boosted antigen-presenting cells (APCs), dendritic cell (DC) activation, M1-type TAMs, and IFN-γ production while reducing suppressive cells [42]. Similarly, the Ag-based liposome LipoAgTNZ increased the infiltration of CD3^+^ and CD8^+^ T cells, reduced CD206^+^ M2 macrophage polarization and recruited more CD44^+^CD62L^+^ memory T cells that provide extended protection against tumor recurrence, with the efficacy dependent on both CD8^+^ and CD4^+^ T cells [14]. Collectively, these results highlight the dual role of nanosystems in suppressing bacteria-induced inflammation and strengthening antitumor immunity, offering long-term anticancer protection.

### 4.2. Safety Evaluation

Since biocompatibility concerns remain the priority issue, especially for nanomaterial-based therapies, a series of biosafety monitoring experiments were carried out, and the detailed evaluation is listed below (Table 4).

#### 4.2.1. In Vitro Assessment

Biocompatibility remains a major challenge for the clinical translation of nanodrugs, and extensive evaluations have been carried out to ensure their safety. Across twelve studies, in vitro biosafety assays were conducted alongside their in vivo assessments to evaluate potential toxicity [25,27,29,30,31,32,36,39,42,43,44,45]. Overall, most nanodrugs exhibited lower toxicity than their free drug counterparts and caused minimal cytotoxicity at therapeutic concentrations, supporting their safety profile for further development. Importantly, nanodrugs also demonstrated enhanced selectivity toward cancer cells lines compared to normal cells. For example, at a concentration of 100 μM, free OXA reduced the viability of mouse fibroblast L929 cells by 60%, whereas OLP/PP nanoassemblies caused only a 20% decrease [30]. Similarly, LTA-MSNs developed by Song et al. showed negligible toxicity to mouse embryonic fibroblasts 3T3 cells while effectively targeting CT26 and M109 cancer cells [29]. Collectively, these findings suggest that nanodrugs not only mitigate off-target toxicity but also preferentially act on malignant cells, strengthening their potential for safe and effective clinical application.

Since most chemotherapies are administered via intravenous infusion [94,95], evaluating the hemolytic activity of nanodrugs is crucial to ensure blood compatibility [96]. Kang et al. demonstrated that the hemolysis threshold (HC_10_, defined as the concentration causing 10% hemolysis) of the amphiphilic polymer SGP_2_ was 5000 µg/mL, which was significantly higher than its therapeutic concentration [39], indicating strong safety margins. Similarly, five additional studies consistently reported negligible hemolytic effects across different nanodrug systems [25,27,29,31,42], further reinforcing their excellent red blood cell compatibility and supporting their suitability for systemic administration.

#### 4.2.2. In Vivo Assessment

In vivo biocompatibility experiments are generally considered more convincing than in vitro assays owing to their ability to reflect complex physiological conditions [97,98]. All selected studies performed in vivo biosafety assessments, with mice serving as the primary experimental models. Among these, fourteen studies employed tumor-bearing mice to evaluate therapeutic toxicity, while ten studies used healthy mice to assess systemic toxicity. Bama minipiglets (*Sus scrofa*), which offer greater physiological similarity to humans [99,100], were used in one study on phage-based therapy [41]. Standard biosafety evaluations include monitoring body weight and performing biochemical blood analysis throughout the experimental period, complemented by hematological profiling in sacrificed animals. Hematological assessments focused on blood cell proportions, whereas biochemical tests measured liver and kidney function markers, such as aspartate transaminase (AST), alanine transaminase (ALT), urea, and creatinine (CREA). In addition, excised organs, including heart, lung, liver, spleen, kidney, and tumor, were subjected to histological examination using hematoxylin–eosin (H&E) and Masson’s trichrome staining. Toxicity was further characterized based on various scoring systems. Although most synthesized nanodrugs demonstrated favorable biosafety profiles in these in vivo evaluations, several noteworthy issues remain and warrant further investigation to fully establish their clinical safety.

### 4.3. Limitations of Preclinical Animal Models in Recapitulating Human Tumor–Microbiome Interactions

Animal models, predominantly murine including xenograft, syngeneic, or orthotopic tumor-bearing mice, provide valuable insights into the in vivo efficacy of nanomaterial strategies against intratumoral bacteria in antimicrobial effects, tumor growth inhibition, and immune modulation [101]. However, these models have inherent limitations that may hinder direct translation to human disease. Compared to implanted animal tumors, human tumors exhibit profound heterogeneity in microbial biomass, spatial distribution, and composition, and murine models frequently involve higher microbial loads or artificial inoculation of specific strains, which may not accurately reflect the low-biomass and contaminant-sensitive nature of human intratumoral microbiota [101,102,103,104]. In addition, mice differ significantly from humans in gastrointestinal physiology, immune system composition, diet, behavior, and microbial community structure [105,106]. These factors can alter how intratumoral bacteria interact with tumor cells, immune infiltrates, and nanomaterials, potentially over- or underestimating therapeutic effects observed in rodents. In addition, while transplanted cell lines or genetically engineered strains are employed in most animal models, they may not fully capture the spontaneous and multifactorial development of human tumors, including chronic inflammation, genetic instability, and long-term microbiome influences [107,108]. This may limit insights into chronic or context-dependent roles of intratumoral microbiota. Moreover, preclinical studies often rely on laboratory-adapted bacterial strains or simplified mono-colonization, which overlook the polymicrobial and dynamic interactions present in human tumors. Environmental factors like housing and diet in animal facilities can introduce variability that does not mirror human exposures.

These limitations underscore the exploratory nature of current preclinical evidence and highlight the need for complementary approaches, such as advanced in vitro models (e.g., tumor organoids or spheroids co-cultured with patient-derived microbiota), humanized mouse models, or multi-omics integration from human cohorts, to bridge the translational gap. Future research should prioritize validation in models that better mimic human intratumoral low-biomass conditions and incorporate patient-derived xenografts or organoids to enhance relevance.

### 4.4. Heterogeneity Across Studies and Implications for Comparability and Future Research

While similar research methodologies were adopted in selected studies, substantial heterogeneity is not neglectable in three key domains.

- Bacterial Strains

A wide range of bacteria, including common pathogens such as *F. nucleatum*, *E. coli*, *P. anaerobius*, and others, were investigated. This diversity reflects different mechanistic roles such as tumorigeneses promoters or therapeutic efficacy suppressors but excludes direct cross-study comparisons of nanomaterial efficacy against specific microbial targets.

- Cancer Models

The reviewed studies employed various preclinical models, including subcutaneous xenografts, orthotopic implants, and chemically or genetically induced models in various mouse strains (e.g., BALB/c, C57BL/6, and nude). Tumor types spanned CRC, breast, pancreatic, cervical, melanoma, lung, and liver cancers. Such variation in tumor microenvironment, vascularization, immune competence, and microbial colonization dynamics influences nanomaterial distribution, bacterial persistence, and therapeutic outcomes, limiting extrapolation across models.

- Outcome Measures

Reported endpoints were highly variable, encompassing bacterial clearance (CFU counts, qPCR), tumor growth inhibition (volume, bioluminescence), survival/progression-free intervals, histological assessments (necrosis, apoptosis), immune profiling (cytokine levels, tumor infiltrating lymphocyte infiltration), and nanomaterial-specific metrics (biodistribution, toxicity). Few studies adopted standardized or core outcome sets, which posed additional challenges for evidence synthesis.

This heterogeneity is typical of a novel research area where exploratory, proof-of-concept studies predominate, but it restricts the ability to draw robust conclusions about the overall effectiveness or optimal strategies of nanomaterial approaches. Consistent with the exploratory purpose of scoping reviews, we have not attempted quantitative pooling but instead presented the evidence narratively, with tabular summaries (Table 2) that explicitly highlight these variations to facilitate assessment. To address the issue of heterogeneity, the need for greater standardization should be emphasized in future works, including consensus on representative bacterial strains, harmonized preclinical models, core outcome sets for intratumoral microbiota nanodrug studies, and reporting guidelines to improve comparability.

### 4.5. Comparative Evaluation of Nanomedicine Platforms

As presented above, five categories of fabricated nanodrug systems were summarized, where all of them were reported to have achieved antibacterial or antitumor efficacy to some extent (Table 2). Although nanotechnologies have been developed for several decades, there are still only a few approved nanodrugs for current clinical applications. Their advantages and limitations should be well considered and addressed.

#### 4.5.1. Passive Targeting Systems

This broad category contains platforms relying primarily on the EPR effect and physicochemical properties for tumor accumulation, without specific ligands for bacteria or cancer cells.

Liposomes have high biocompatibility, efficient encapsulation of both hydrophilic and hydrophobic cargos, tunable sizes for EPR optimization, and low immunogenicity. Such advantages enable them to be selected as one of the approved formulations due to their biosafety. Their amphiphilic structure makes liposomes able to be successfully loaded with antimicrobials or chemotherapeutics. Consequently, liposomes are excellent for initial proof-of-concept antimicrobial delivery or combination therapies in accessible tumors. However, the relatively poor deep tumor penetration in hypoxic regions rich in intratumoral bacteria, potential instability in serum due to the formation of protein corona, and limited intrinsic antimicrobial activity hamper their translation [109]. 

Polymeric nanocarriers like PLGA-based polymers often form self-assembled nanomicelles that have tunable degradation for sustained release, good stability, and flexibility for co-delivery, but when it comes to antibacterial efficacy, their slower release kinetics may reduce rapid bacterial killing. Additionally, their degradation products may cause potential inflammation, and variable charges and sizes may affect penetration [110]. Taken together, polymer-based nanodrugs are best for long-term microbiota modulation and sustained antitumor effects.

Apart from organic nanoplatforms, some inorganic nanosystems (e.g., silver, iron oxide, and silica) have also been introduced. This type of nanosystem generally produces potent ROS and metal ions that induce strong intrinsic antimicrobial effects. With specific components, some inorganic nanosystems can realize multiple functions of both imaging and therapy and have high stability. In contrast, metal accumulation may raise concerns about potential cytotoxicity, immunogenicity, and challenges in selective bacterial or host cell targeting [111]. As a result, such a strategy is highly effective for direct bacterial eradication in resistant settings but requires surface modification for safety.

Drug nanoassemblies consist of self-assembled prodrugs or pure drug nanoparticles. They have the following advantages, such as high drug loading, minimal excipients, and simple preparation [112]. On the other hand, for the selected publication where MTI–FDU nanoassemblies were designed, as the two major drugs are an antibiotic and a chemotherapeutic agent, there were possible limitations of targeting specificity, stability issues, and variable pharmacokinetics [28,112]. Therefore, drug nanoassemblies are useful for high-payload antimicrobial delivery but less ideal for complex intratumoral environments.

#### 4.5.2. Active Targeting Drug Delivery Systems

Active targeting drug delivery systems incorporate ligands or bio-inspired designs for specific recognition of bacteria or cancer cells and thus can achieve better targeting.

For direct bacterial targeting and cancer cell targeting approaches, bacteria- or tumor-specific ligands tend to be embedded on the nanoparticles. The specific binding between ligands and receptors enhances specificity, reduces off-target effects, and improves efficacy against specific strains. As such nanosystems may introduce foreign proteins, potential immunogenicity may arise. The ligand–receptor variability across tumors and bacteria is also a knotty issue and is likely to give rise to complex synthesis [113]. Nevertheless, this strategy is ideal for precision elimination of pathogenic intratumoral bacteria while sparing beneficial ones.

Bacterial membrane-mimicking strategies refer to bacterial membrane-coated NPs. Similar to the above direct targeting methods, the modified bacterial membrane shells have the property of natural tumor tropism (e.g., hypoxia-seeking). In addition, the antigens on the membrane surface may trigger synergistic immunomodulation. The most obvious problem of the strategy is elevated safety risks. If not properly prepared, the adopted bacterial membrane may have residual immunogenicity and even cause sepsis. Owing to the complexity of manufacturing, the scalability challenges remain [114,115]. Ultimately, regulatory issues for bio-hybrids should also be under consideration. Therefore, this approach is particularly promising for deep tumor penetration and combined antimicrobial–immunotherapeutic effects.

#### 4.5.3. Phototherapy

PDT depends on light-triggered ROS generation for bacterial killing and tumor ablation. The administration process enables spatiotemporal control. The disadvantages include limited tissue penetration of light and oxygen dependence. Therefore, the therapy may fail in hypoxic cores. Another concern is photosensitizer toxicity that may prevent its promotion [116]. Given the circumstances, PDT is effective for surface-accessible tumors or in combination with bacteria-targeting carriers.

Another phototherapy is PTT that achieves direct thermal ablation of bacteria and tumors. Thanks to the light source of NIR, the therapy can realize deep tissue heating. It also exhibits synergy with nanomaterials like gold NPs. The flaws mainly fall on the specificity; non-specific heating or overheating may damage normal tissues [116]. PTT is thus suitable for local control in bacteria-colonized tumors.

#### 4.5.4. Phage Therapy

Phages have been attracting significant attention since the growing antibiotic-resistant bacterial strains emerged. Phages have high specificity to bacterial strains, and the nature of self-amplification dramatically increases the administration intervals. Their low toxicity to hosts make them a seemingly ideal choice for bacterial suppression. However, a narrow host range, potential phage resistance, and delivery challenges are obstacles before they obtain recognition from physicians [117]. Currently, phages are most promising in targeting specific intratumoral pathogens like the widely reported tumor-promoting *F. nucleatum* in CRC. Some scientists conjugated phages with NPs to give phage–nanocomposites that combine phage specificity with nanomaterial multifunctionality for enhanced stability and drug delivery. Like bacterial membrane-mimicking strategies, the complex assembly and potential immunogenicity of phage–nanocomposites may hamper their advancement [118]. Still, phage-based therapy provides potential for precision bacterial targeting with added therapeutic payloads.

#### 4.5.5. Other

Nanozymes offer intrinsic catalytic generation of ROS or O_2_ to overcome hypoxia. They show high stability; low cost; and multifunctionality including modulating tumor metabolism, inducing ferroptosis or pyroptosis, or selectively eradicating pathogenic intratumoral bacteria but may suffer from limited catalytic efficiency in low-H_2_O_2_ tumor environments, potential off-target ROS toxicity, and challenges in precise bacterial selectivity [119].

Ultrasound entitles SDT to provide deep tissue penetration with non-invasiveness and spatiotemporal control, as well as reduced skin phototoxicity compared to PDT or PTT. As SDT relies on sonosensitizer accumulation and oxygen, variable ultrasound levels in heterogeneous tumors may be observed, and emerging safety data for repeated use are not sufficient [120]. The strategy is highly promising for hypoxic or deep-seated tumors with abundant intratumoral bacteria.

To summarize, since each nanomedicine platform has its strengths and limitations, no single approach can fully meet all the complicated requirements in targeting intratumoral microbiota, such as deep penetration into hypoxic regions, high specificity to pathogenic bacteria, potent antimicrobial and antitumor effects, excellent biocompatibility, minimal off-target toxicity, and admirable translational feasibility. The optimal strategy probably lies in developing hybrid therapies that integrate the complementary advantages of different platforms while decreasing their individual drawbacks. For instance, combining bacterial membrane-mimicking or phage-based active targeting with nanozymes has shown promise in preclinical models for synergistic antimicrobial and immunomodulatory effects [121]. Another example is the integration of phototherapy or SDT with passive targeting carriers or bacteria–nanoparticle hybrids, which enable enhanced tumor ablation, bacterial eradication, and hypoxia alleviation while reducing light penetration limitations of pure PDT or SDT [122].

### 4.6. Mechanistic Insights into Overcoming Bacteria-Induced Chemoresistance

The included studies mainly dealt with chemotherapy resistance conferred by intratumoral *E. coli* [24,37], *F. nucleatum* [26,30,33,34,41,44], and *B. fragilis* [10]. They collectively demonstrated that nanomedicine platforms overcome bacteria-induced chemoresistance primarily through selective depletion or functional modulation of key intratumoral pathogens.

For *F. nucleatum*, the clearance disrupts autophagy and TLR4/NF-κB-mediated protective signaling, as evidenced by reduced autophagosome formation and restored drug-induced apoptosis [7,123]. The elimination of *E. coli* alleviates gemcitabine inactivation via cytidine deaminase-mediated metabolism to the inactive metabolite 2′,2′-difluorodeoxyuridine (dFdU), thereby reinstating gemcitabine’s cytotoxic efficacy in breast and pancreatic models [8]. Similarly, reduction of *B. fragilis* attenuates chronic inflammatory STAT3 activation, decreasing anti-apoptotic protection [10].

These mechanisms are supported by consistent molecular readouts (e.g., Western blot for LC3/p62, cleaved caspase-3, and p-STAT3; qPCR for inflammatory cytokines; and TUNEL assay for apoptosis) across multiple preclinical models. Nevertheless, the evidence remains predominantly from in vitro co-cultures and murine models; direct causality in human low-biomass tumors awaits further validation through multi-omics approaches and patient-derived models.

### 4.7. Selectivity of Nano-Based Systems for Intratumoral Pathogenic Bacteria

Several included studies relied on passive targeting via EPR effect; size or charge properties; or broad-spectrum antimicrobial loads like antibiotics, silver ions, and ROS-generating nanozymes, which lack strong selectivity for intratumoral versus commensal bacteria and thus raise concerns about potential damage to beneficial microbiota, particularly in the gut or tumor-adjacent tissues. Differently, several strategies reported in the reviewed literature showed early promise for improving specificity. Ligand-based active targeting methods such as LTA-MSN-targeting bacterial lipoteichoic acid [29], OLP/PP nanoassembly with sialic acid-targeting phenylboric acid [30], or HA@Met-f-ZIF_D_ nanogels containing HA, with the ligand of HAase overexpressed in cancer cells [31], and PG-Pt-LA/CB[7] assembly where CB[7] is disassembled by overexpressed spermine in the tumor microenvironment [32] enable preferential binding to the target bacterium-enriched tumors. The bacterial membrane-mimicking approach targeting *F. nucleatum* [33,34] or *P. anaerobius* [36] also leads to preferential accumulation in the corresponding bacteria-colonized tumor sites. Experimental data display minimal impact on gut microbiota composition using 16S rRNA sequencing compared to non-treated counterparts. Tumor microenvironment-responsive designs include stimuli-responsive nanozymes [44], SDT [45], and PDT [37] exhibiting enhanced activity in the acidic, hypoxic, and ROS-rich intratumoral niche where pathogenic anaerobes predominate. While not strictly species-specific, these designs inherently favor bacteria prospering in tumor-specific conditions over commensals in normoxic tissues.

Despite these advances, true species-level or intratumoral-exclusive specificity remains limited. Most studies do not perform comprehensive off-target assessments (e.g., longitudinal 16S sequencing of gut/fecal microbiota or multi-site tissue analysis). Broad-spectrum antimicrobials (e.g., colistin in some systems) can affect commensal Gram-negative bacteria. Clinical translation requires the apparent evaluation of microbiome impact, especially given the low-biomass nature of human intratumoral communities and the risk of dysbiosis-related complications. To address these gaps, future nano-based platforms should prioritize high-affinity, species-specific ligands (e.g., aptamers, engineered phages, or synthetic receptors against unique bacterial surface epitopes) and incorporate microbiome-safe drug loads (narrow-spectrum antibiotics or ROS-generating systems with tumor-restricted activation). Preclinical evaluation using humanized microbiota models, germ-free mice colonized with patient-derived flora, and longitudinal metagenomic profiling are essential to confirm intratumoral selectivity and minimize commensal disruption.

### 4.8. Translational Challenges of Multifunctional Nanomedicines

#### 4.8.1. Scalability, Regulatory Complexity, and Reproducibility

The reviewed nanomedicine strategies, while demonstrating promising preclinical efficacy against intratumoral microbiota and associated chemoresistance, face significant translational hurdles that remain underexplored in most of the included studies. A significant limitation of the current evidence base is the limited attention to translational feasibility. Most studies are proof-of-concept and do not address scalability, GMP-compliant manufacturing, batch-to-batch reproducibility, or regulatory classification, which are key determinants of whether these approaches can progress beyond the preclinical stage [124,125].

To bridge the translational gap, future research should emphasize: (1) process development and scale-up studies to improve reproducibility and manufacturability; (2) early regulatory engagement to clarify classification; (3) standardized characterization protocols and inter-laboratory validation; and (4) incorporation of translational endpoints (e.g., cost-of-goods estimates, stability studies under accelerated conditions, and microbiome safety profiling) in preclinical pipelines. These steps will be essential to move promising multifunctional nanomedicines from bench to bedside.

#### 4.8.2. Theranostic Applications

Platforms combining antimicrobial drug loads with imaging capabilities generally include the following three categories. First is the fluorescent or phototheranostic systems such as porphyrin- or black phosphorus-based nanoparticles for simultaneous fluorescence imaging, PDT/SDT, and ROS-mediated bacterial killing [116]. The second type is magnetic nanozymes. For example, Fe_3_O_4_-based or single-atom nanozymes with T_2_-weighted MRI contrast and catalytic ROS generation for chemodynamic therapy and bacterial eradication [119]. The last one is phage-nanocomposites: the M13 phage conjugated with gold nanorods or quantum dots for targeted bacterial imaging (via fluorescence or photoacoustic signals) [118]. These designs enable “see-and-treat” paradigms, where bacterial infection is visualized to guide treatment decisions and then therapeutically addressed in the same platform.

While preclinical proof-of-concept exists, clinical translation of theranostic nanosystems faces hurdles. The low sensitivity for detecting low-biomass intratumoral infections requires ultra-sensitive probes. Potential off-target accumulation in commensal-rich sites may induce undesired side effects. Regulatory complexity for combined diagnostic–therapeutic agents should also be considered. Future directions should focus on bacteria-specific, high-sensitivity imaging probes (e.g., smart probes that are only activated upon bacterial binding) and early-phase trials evaluating theranostic efficacy in biomarker-enriched cohorts.

### 4.9. Integration of Antibacterial Nanomedicine into Current Cancer Regimens

The reviewed preclinical evidence offers several plausible integration points for nano-based antibacterial strategies within current regimens, particularly in cancers with well-documented intratumoral microbiota involvement (e.g., colorectal, pancreatic, and breast cancers) [126].

Adjuvant chemotherapy: Short-course targeted nano-antibacterial therapy could be administered before or concurrently with standard regimens (e.g., FOLFOX/FOLFIRINOX in colorectal or pancreatic cancer or anthracycline/taxane in breast cancer) to deplete key intratumoral bacteria (e.g., *F. nucleatum* and *E. coli*) and potentially restore chemosensitivity [34].

Immunotherapy combinations: In checkpoint inhibitor-eligible cancers, an initial antibacterial nanodrug cycle could accompany anti-PD-1/PD-L1 agents to enhance T-cell infiltration and convert immunologically “cold” tumors, similar to current chemo-immunotherapy schedules [127].

Maintenance or relapse settings: They act as a low-toxicity adjunct to maintenance therapy (e.g., capecitabine, PARP inhibitors) to delay or prevent microbiota-driven resistance recurrence [101].

Lack of direct evaluation of combination feasibility, interaction toxicity, dosing schedules, or biomarker-driven patient selection with standard-of-care regimens remains a key limitation. Future efforts should focus on biomarker-enriched early-phase platform trials testing antibacterial nanomedicines as supplements of standard regimens, with co-evaluation of microbiome safety, optimal sequencing, and combination endpoints.

### 4.10. Highlights and Limitations

Recent advances in nanotechnology have opened exciting avenues for targeting intratumoral bacteria, which is a previously underdetermined contributor to cancer progression, therapeutic resistance, and infection-related complications. The nanosystems discussed here have demonstrated promising efficacy in reducing the intratumoral bacterial burden, reshaping the tumor microbiome and restoring sensitivity to conventional chemotherapy, as well as novel immunotherapy. Beyond microbial clearance, many of these platforms also modulate the immune systems within the TME, offering multifaceted benefits for cancer management.

This scoping review offers a comprehensive analysis of the included nano-based therapeutic strategies. While CRC-associated *F. nucleatum* dominates as the most frequently targeted species and cancer type, multiple other bacterial species (*E. coli*, *S. aureus*, and *B. fragilis*) were investigated across various cancer types (Table 2), highlighting the relevance of intratumoral bacteria beyond a single bacterial species and malignancy. The diversity of nanosystem designs also reflects the complexity of tumor–microbiome interactions and highlights the need for tailored context-specific therapeutic solutions.

It is also worth noting that intratumoral bacteria are not only associated with tumor progression but also contribute to severe local and systemic infections, which are a leading cause of cancer-related mortality [44,128]. Immunocompromised cancer patients are particularly vulnerable to bacterial infections. The TME provides a unique survival niche for pathogenic bacteria, enabling them to evade immune surveillance, resist antibacterial treatments and persist within tumor tissues. This persistence may lead to recurrent infections and disruption of cancer treatments [4]. Although the included studies did not primarily focus on this aspect, nanosystems capable of selectively eliminating pathogenic bacteria, like *E. coli* and *S. aureus*, within tumors may serve a dual therapeutic role by both enhancing cancer treatment outcomes and mitigating infection-related complications.

Although the available preclinical findings are promising, the exploration of nano-based therapies for targeting intratumoral bacteria is still in its infancy. Several challenges must be addressed before these promising strategies can be translated into clinical practice. Firstly, the heterogeneity of intratumoral bacteria across patients and cancer types may make the design of universally effective targeting nanosystems almost impossible. Personalized profiling of tumor-associated microbiota to guide therapeutic selection may be a more feasible approach. Secondly, concerns about off-target effects, long-term biosafety of the nanosystems and their degraded products, and potential disruption of beneficial microbiota must be rigorously evaluated both preclinically and clinically. Thirdly, most current studies rely on murine models, which may not fully recapitulate human tumor–microbiome interactions. The development of humanized models and organoid systems to provide better predictions of clinical outcomes in humans are critical for their successful translation. Lastly, the complexity of nanosystem fabrication, including scalability, reproducibility, and regulatory compliance, also presents significant translation barriers and requires significant research efforts.

Moving forward, establishing standardized protocols for microbiome characterization, nanosystem evaluation, and therapeutic efficacy assessment will be essential to align multidisciplinary research efforts and accelerate the clinical translation of prospective nanoplatforms. As our understanding of the tumor microbiota improves, intratumoral bacteria-targeting nanomedicine may evolve into a cornerstone of precision oncology and infection control, offering hope for patients with resistant, microbiota-influenced, or infection-prone malignancies.

## 5. Conclusions

Emerging evidence confirms the presence of intratumoral bacteria, and their roles in tumorigenesis, tumor progression and modulation of therapeutic responses are increasingly being recognized [4]. This growing understanding has opened a novel avenue for precise targeting in cancer therapy. In conjunction with the advancement in nanotechnologies, the development of multimodal treatment strategies capable of simultaneously eradicating cancer and bacterial cells is being increasingly explored. This scoping review outlined the therapeutic promise of nanosystems designed to target intratumoral bacteria, and their potential to reduce bacterial burdens and enhance tumor sensitivities to chemotherapy, immunotherapy, and host immune surveillance were well demonstrated. The diversity of bacterial species, cancer types, nanosystem designs, and efficacy evaluation protocols investigated reflects the complexity of this emerging field and highlights the need for tailored approaches to optimize the clinical translation of promising candidates.

## Figures and Tables

**Table 1:** Inclusion and exclusion criteria.

Category	Sub-Type	Criteria
Inclusion criteria	Population	Studies involving tumor-associated (intratumoral) pathogenic bacteria within the tumor microenvironment
	Concept	Nanodrug strategies designed for antimicrobial effects against intratumoral bacteria or for modulating bacteria to achieve antitumor outcomes (e.g., targeted killing of bacteria-infected tumor cells, bacteria-mediated drug delivery, or combating both bacteria and tumors for enhanced antitumor effects)
	Context	Preclinical studies in cancer/tumor settings, including in vitro antimicrobial studies (e.g., bacterial killing or inhibition assays in tumor cell co-cultures) and in vivo anticancer efficacy studies in bacteria-infected tumor-bearing animal models (e.g., tumor growth inhibition, survival, or immune response assessments)
	Study types	Original primary research articles (experimental, preclinical)
	Language	English
	Publication date	No restrictions (from database inception to the date of the final search)
	Publication status	Peer-reviewed published articles on recognized platforms with DOI
Exclusion criteria	Study types	Clinical studies in humans, case reports, reviews, editorials, commentaries, letters, or protocols without original data
	Study content	Studies exclusively adopting probiotics or engineered bacteria as an antitumor approach
		Studies on tumor-associated microbiota that do not involve nanomaterials/nanodrugs (e.g., purely descriptive microbiome profiling or non-nano interventions)
		Studies using nanomaterials for cancer therapy without any focus on or interaction with intratumoral microbiota/bacteria
	Language	Non-English publications
	Availability	Studies for which full texts could not be retrieved after exhaustive efforts

**Table 2:** Summary of the assessment of the nanodrugs in the selected studies.

Study	Formulation/Targeting Strategy	Cancer Type	Bacterial Strain	Animal Model	Infection Method	Nanosystems Design	Key Findings
Passive targeting							
Wang et al. (2024) [14]	Liposomes	CRC	*F. nucleatum* *E. coli* Nissle	1. Female BALB/cJ mice (1) CT26(FL3)-RFP/Luc (orthotopic) (2) Liver metastasis model 2. Female C57BL/6J mice MC38 (orthotopic)	i.g.	Antibiotic silver–tinidazole complex encapsulated in liposomes (LipoAgTNZ)	No bioluminescence change of tumor by eradicating gut colonizing *F. nucleatum* with polymyxin B emphasized the role of tumor colonizing *F. nucleatum* on tumorigenesis LipoAgTNZ did not cause gut microbiome dysbiosis LipoAgTNZ elicited antitumoral immune response
Wang et al. (2023) [23]	Liposomes	Liver CRC Breast	*E. coli* Xen14	Female BALB/c mice HCT116 (s.c.)	i.t.	Self-targeted DCPA-H_2_O liposomes for the co-delivery of gemcitabine and ciprofloxacin (GC-DCPA-H_2_O)	Two gemcitabine metabolites produced by *E. coli* were identified: cytosine and N-acetyl-2′-deoxy-2′,2′-difluorocytidine pH-responsive DCPA-H_2_O liposomes made of complexed water achieved better treatment outcome compared with non-self-targeting liposomes or free drugs
Qiu et al. (2022) [24]	Drug nanoassembly	Breast	*E. coli* Nissle 1917	Female BALB/c mice 4T1-Luc (s.c.)	i.v.	Colistin crosslinked gemcitabine micelle (CCGM)	CCGM prevented gemcitabine from being metabolized by intratumor *E. coli*, overcoming chemoresistance
Li et al. (2024) [25]	Polymer	CRC	*F. nucleatum*	Male BALB/c mice CT26 (s.c.)	i.v.	A nanodrug (O-SPIONs@PG-Pt-LA, OPPL) integrating oleic acid-modified superparamagnetic iron oxide nanoparticles (O-SPIONs) and amphiphilic polymer (poly(glycidol)-OxPt-COOH-lauric acid, PG-Pt-LA) to deliver the platinum prodrug and antimicrobial lauric acid (LA)	OPPL synergistically enhanced antibacterial and biofilm disruption activities against *F. nucleatum* by producing ROS through its peroxidase-like activity OPPL increased intracellular reactive oxygen species (ROS), promoted lipid peroxides and depleted glutathione, leading to ferroptosis. OPPL enabled in vivo magnetic resonance imaging
Chen et al. (2023) [26]	Inorganic	CRC	*F. nucleatum* (ATCC 25586)	Male BALB/c nude mice HCT116 (s.c.)	i.t.	Dendritic mesoporous silica NPs combining antibacterial Ag and antitumor drug epirubicin (Ag@DMSNs-EPI NPs, ADEN)	ADEN blocked *F. nucleatum*-induced autophagy, overcoming chemoresistance
Chu et al. (2022) [27]	Inorganic	Cervical Breast	*E. coli* *S. aureus*	Female BALB/c nude mice 4T1-Luc (s.c.)	s.c. on surgical site	Fluorescent flavonoid-silica nano composites (FSiNCs) incorporated with fibrinogen nanogel (FSiNCs@Fibrin)	FSiNCs induced specific and selective accumulation of ROS in tumors and bacteria cells FSiNCs@Fibrin inhibited post-surgical tumor recurrence and bacterial infection
Gao et al. (2023) [28]	Drug nanoassembly	CRC	*F. nucleatum* (ATCC 10593)	1. C57BL/6 mice AOM–DSS induced 2. Female BALB/c mice CT26 (s.c.)	1. i.g. 2. i.t.	Metronidazole–fluorouridine linked by disulfide bond to form self-assembled nanoparticles (MTI–FDU) that are glutathione (GSH)-responsive	MTI–FDU had minimal effect on gut microbial homeostasis and maintained the microbiota diversity MTI–FDU shaped the tumor immune microenvironment through clearance of bacteria and bacterial products
Active targeting							
Song et al. (2022) [29]	Targeting bacteria	CRC Lung	*P. anaerobius* (GIM1.536) *S. aureus* (ATCC 25923)	BALB/c mice (1) CT26-Luc (orthotopic) (2) CT26 (s.c.)	(1) Uninfected (2) i.t.	Bacteria-targeting mesoporous silica nanoparticles decorated with bacterial lipoteichoic acid antibody (LTA-MSNs) loading anticancer drug irinotecan (IRT) (LTA-MSN@IRT), and the MSNs decorated with DBCO for targeting bacteria through bio-orthogonal reactions (DBCO-MSN@IRT)	LTA-MSNs could precisely target bacteria in tumors and deliver antitumor drugs
Li et al. (2023) [30]	Targeting tumor	CRC	*F. nucleatum* (ATCC 25586)	Female BALB/c nude mice HT-29 (s.c.)	i.v.	Antibacterial lauric acid (LA) and sialic acid-targeting phenylboric acid (PBA) were first conjugated to oligomethyleneimine (OEI) to form OEI-LA-PBA (OLP) followed by interacting with platinum(IV) oxaliplatin prodrug (OXA-COOH)-modified polyglycidyl ether (PG) (PG-OXA-COOH, PP) to construct the pH- and GSH-responsive OLP/PP nanoassembly	OLP/PP had no toxicity to *C. butyricum*, and limited toxicity to *E. coli* OLP/PP overcame bacteria-induced OXA chemoresistance
Xie et al. (2024) [31]	Targeting tumor	CRC	*F. nucleatum* (ATCC 25586)	BALB/c nude mice HCT116 (s.c.)	i.t.	HA@Met-f-ZIF_D_ nanogels (NGs): zinc-imidazolate frameworks (ZIF) with doxorubicin (DOX) loading and folate grafting (f-ZIF_D_) mixed with metronidazole (MET) and encapsulated in NGs crosslinking sulfhydryl HA (HA-SH), sulfhydryl sodium alginate (SA-SH) and 4-arm polyethylene glycol acrylate (PEG-Acr)	NGs are HAase and acid dual responsive NGs achieved size tunability and cascaded release of MET and DOX NGs significantly enhanced bioavailability and increased half-lives of MET and DOX by around 20 times NGs treatment confirmed the synergy of MET and DOX
Yan et al. (2022) [32]	Targeting tumor	CRC	*F. nucleatum* (ATCC 25586)	1. Male BALB/c nude mice HT-29 (s.c.) 2. BALB/c mice CT26-Luc (orthotopic)	1. i.v. 2. i.g.	Conjugating LA and platinum (IV) oxaliplatin prodrug (OxPt-COOH) to hyperbranched poly(glycidol) (PG), followed by addition of cucurbit[7]uril (CB[7]) disassembled by overexpressed spermine in tumor microenvironment to elicit supramolecular assembly (PG-Pt-LA/CB[7])	PG-Pt-LA/CB[7] reversed *F. nucleatum*-induced autophagy Dual response: spermine led to the size change of NPs; GSH conversed a Pt(IV) prodrug to an active Pt(II) species
Chen et al. (2023) [33]	Bacterial membrane-mimicking	CRC Melanoma	*F. nucleatum* (ATCC 25586)	1. Female BALB/c mice CT26 (s.c.) 2. Female C57BL/6J mice (1) AOM–DSS induced (2) MC38 (s.c.)	1. i.t. 2. (1) i.g. (2) i.t.	Fusing *F. nucleatum* cytoplasmic membrane (FM) with colistin-loaded liposomes (Colistin-Lipo) through extrusion to obtain Colistin-loaded FM-fused liposomes (Colistin-LipoFM)	The attachment of *F. nucleatum* to CRC cells is dependent on its binding of Fap-2 with Gal-GalNAc Colistin-LipoFM only targeted *F. nucleatum* but not microbiota Colistin-LipoFM enhanced immunotherapy by overcoming immune resistance induced by *F. nucleatum* infection by elevated MDSCs and reduced CD8^+^ T cells
Chen et al. (2024) [34]	Bacterial membrane-mimicking	Breast	*F. nucleatum* (ATCC 25586) *E. faecalis* (ATCC 29212) *S. sanguis* (ATCC 10556) *S. xylosus* (ATCC 29971)	Female BALB/c mice (1) 4T1 (s.c.) (2) Lung metastasis of 4T1-Luc (orthotopic) (3) 4T1-Luc (orthotopic)	i.t.	Nanovehicles fusing the extracted *F. nucleatum* cytoplasmic membrane (FM) containing Fap-2 with antibiotic-loaded liposomes (Antibiotic-LipoFM)	*F. nucleatum*-conferred chemoresistance was confirmed in both clinical samples and in vitro and in vivo experiments from decreased caspase-3 and upregulated autophagy Antibiotic-LipoFM overcame bacteria-induced chemoresistance and inhibited tumor metastasis
Han et al. (2023) [35]	Bacterial membrane-mimicking	Lung	*E. coli* *S. aureus* *S. Intermedius* *P. intermedia*	Female BALB/c mice (1) Bilateral M109 (s.c.) (2) Unilateral M109 (s.c.) (3) M109-Luc (i.v.) (4) Urethane induced	i.t.	Capsular polysaccharide (CP)-camouflaged gallium-polyphenol metal–organic network (MON) (GaTa-CP NPs) loaded with etoposide (GaTa-CP@Eto NPs)	Microbiota-induced chemoresistance via the overexpression of P-gp on tumor cells was revealed CP coating reduced cell uptake and thus allows long-term retention Local lung microbiota diversity remained after treatment GaTa-CP@Eto NPs abrogated drug resistance by bacteria clearance and down-regulated P-gp
Zhuang et al. (2023) [36]	Bacterial membrane-mimicking	CRC	*P. anaerobius* (ATCC 27337) *F. nucleatum* (ATCC 25586)	BALB/c mice (1) CT26 (s.c.) (2) CT26-Luc (orthotopic)	(1) i.t. (2) i.g.	Biomimetic nanovaccine *P. anaerobius*-MnO_2_-OXA (PMO) through biomineralization to load MnO_2_ and OXA on the surface of inactivated *P. anaerobius* that has a natural affinity to CRC	PMO increased probiotics, decreased tumor-promoting bacteria, and restored microbiota diversity PMO remodeled tumor immune microenvironment and prevented tumor recurrence
Phototherapy							
Liu et al. (2024) [37]	PDT	Pancreatic	*E. coli* Nissle 1917 (EcN)	BALB/c nude mice Panc02 (s.c.) mixed with sfGFP- and luxCDABE-labeled EcN	Mixed with cells	Antimicrobial peptide (UBI29–41) linked with a chlorophyll-derived photosensitizer pyropheo-phorbide-a (Ppa) by a monodisperse poly(ethylene glycol) (PEG) chain to form amphiphilic conjugates (Ppa-PEG-UBI), which self-assemble into micelles (UPPM) in aqueous solution	UPPM did not affect *E. coli* in gut UPPM reversed gemcitabine resistance induced by *E. coli*
Kong et al. (2022) [38]	PTT	Breast CRC	*E. coli* (ATCC25922) *S. aureus* (ATCC6538)	BALB/c mice (1) 4T1 (s.c.) (2) CT26 (s.c.)	Fecal environment	Photothermal agent Nb_2_C nanosheets (NSs) as support to anchor Au nanoparticles (NPs) and accommodate anti-TNF-α drug (Nb_2_C/Au/anti-TNF-α-PVP)	The phototherapy altered the abundance and diversity of intratumoral microbiomes The phototherapy synergistically mitigated bacterial-induced inflammation, and disrupted the metabolism of intratumoral microbiota and tumor microenvironment, thus unfreezing tumor resistance
Kang et al. (2022) [39]	PTT	Pancreatic	*E. coli* Nissle 1917	Male C57BL/6 mice Pan02 (s.c.)	i.v.	Dual-cascade responsive nanoparticle (sNP@G/IR) with hyaluronic acid (HA) shell and GSH-responsive polymer-core (NP@G/IR) encapsulating gemcitabine (Gem) and photothermal agent (IR1048)	sNP@G/IR overcame bacteria-mediated Gem inactivation, and activated tumor immunity
Phage therapy							
Ding et al. (2025) [10]	Native phage	CRC	*B. fragilis* (ATCC 43860)	1. Male NU/J mice HT29 (s.c.) 2. Male C57BL/6J mice AOM–DSS induced	1. i.t. 2. i.g.	*B. fragilis*-specific phage VA7	Systematically investigated and identified *B. fragilis* as a chemoresistance promoter in CRC by activating host Notch1 signaling pathway through SusD/RagB *B. fragilis* also decreases the expression of pro-apoptosis proteins like cleaved caspase-9 and PARP, and promotes EMT process *B. fragilis* compromised 5-FU/OXA efficacy in CRC cells and in mouse models Phage VA7 eliminated *B. fragilis* and restored chemosensitivity of CRC in mice
Lam et al. (2025) [40]	Native phage	CRC	*F. nucleatum* 34597 (clinical isolate)	Male BALB/c mice HCT-116 (s.c.)	Mixed with cells	*F. nucleatum*-specific phage ØTCUFN3	ØTCUFN3 inhibited CRC proliferation and the expression of EMT markers in *F. nucleatum*-induced CRC cell lines, p53^+/+^, and p53^−/−^ isogenic HCT116 cells ØTCUFN3 has good biosafety
Zheng et al. (2019) [41]	Phage–nanocomposite	CRC	*F. nucleatum* (ATCC 10953)	1. BALB/c mice (1) CT26 (s.c.) (2) CT26-Luc (orthotopic) 2. Male C57BL/6J *Apc*^Min/+^ mice Genetically induced	1. (1) i.t. (2) i.g. 2. i.g.	Irinotecan (IRT)-loaded dextran nanoparticles (IDNPs) covalently linked to azide-modified phages P2 (A-phages) specific to *F. nucleatum* (IDNP–A-phage)	Tumor-promoting effect of *F. nucleatum* and tumor inhibition effect of the metabolite butyrate of *C. butyricum* were validated The mechanism of *F. nucleatum*-induced chemoresistance was investigated: upregulation of anti-apoptosis genes and down-regulation of autophagy IDNP–A-phage promoted the proliferation of the anticancer probiotic *C. butyricum* IDNP–A-phage exhibited biosafety, and specifically targeted *F. nucleatum* IDNP–A-phage reversed chemotherapy resistance
Dong et al. (2020) [42]	Phage–nanocomposite	CRC	*F. nucleatum* (ATCC 10953)	Female BALB/c mice (1) CT26-Luc (orthotopic) (2) CT26 (s.c.)	(1) i.g (2) i.t.	Silver nanoparticles (AgNP) assembled electrostatically on the surface capsid protein of a temperate and specifically *F. nucleatum*-binding M13 phage (M13@Ag)	M13@Ag increased antitumor bacteria *Butyricicoccus* M13@Ag increased microbiota abundance M13@Ag reversed immunosuppressive TME: reduction in MDSC amplification, activated APCs M13@Ag had synergy with chemo/immuno therapy
Zhao et al. (2025) [43]	Phage–nanocomposite	CRC	*S. gallolyticus* BNCC 188152	Male C57BL/6J mice AOM–DSS induced	i.g.	DNA nanopatches (DNPs) composed of DNA origami nanosheets and phage capture strands were integrated with *S. gallolyticus*-targeted phages (P-Sg) to form DNA nanopatch-bacteriophage system (DNPs@P) via the biolinker of sulfosuccinimidyl-4-(Nmaleimidomethyl) cyclohexane-1-carboxylate (sulfo-SMCC), and then encapsulated with an enteric polymer acrylic resin (L100-55) to obtain DNPs@P-L	*S. gallolyticus* was identified as a promoter of colitis-associated colorectal cancer (CAC) together with inflammatory bowel disease (IBD) DNPs@P-L showed good biosafety DNPs@P-L scavenged ROS to reduce oxidative stress damage to tissues DNPs@P-L restored gut microbiota, and reduced pathogenic *Proteobacteria*, while increased probiotics *Lachnospiraceae*
Other							
Wang et al. (2023) [44]	Nanozyme	CRC	*F. nucleatum*	BALB/c nude mice HCT116 (s.c.)	i.t.	Protein-supported copper single-atom nanozyme (BSA-Cu SAN)	BSA-Cu SAN could generate ROS and deplete GSH The resistance of CRC through elevated autophagy mediated by *F. nucleatum* was relieved by BSA-Cu SAN BSA-Cu SAN is biocompatible as it can be thoroughly cleared by kidney
Qu et al. (2023) [45]	SDT	CRC	*F. nucleatum* (ATCC 25586)	Male BALB/c nude mice (1) HCT116 (s.c.) (2) HCT116-Luc (orthotopic) (3) Lung metastasis of HCT116-Luc (i.v.)	(1) i.t. (2) i.g. (3) pre-infection	Bovine serum albumin (BSA) entraping Au nanoparticles (Au NPs) and modified with an antibacterial metalloporphyrin (CuPpIX) sonosensitizer (Au@BSA-CuPpIX)	Au@BSA-CuPpIX reduced the phototoxicity of sonosensitizer accumulated in the skin Au@BSA-CuPpIX produced ROS under ultrasound Au@BSA-CuPpIX reduced the levels of apoptosis inhibiting proteins by inhibiting intratumoral *F. nucleatum*, thereby enhancing ROS-induced apoptosis Au@BSA-CuPpIX inhibited lung metastasis

**Table 3:** Examples of specific targeting molecules used in nano-based systems for intratumoral microbiota or cancer cell targeting.

Category	Study	Nanoplatform	Targeting Ligand/Molecule	Target
Active targeting	Song et al. (2022) [29]	Mesoporous silica nanoparticles	Lipoteichoic acid antibody	Gram-positive bacteria
	Li et al. (2023) [30]	Nanoassembly	Phenylboric acid	Sialic acid epitope in CRC cells
	Xie et al. (2024) [31]	Zinc-imidazolate framework (ZIF) nanogels	Hyaluronic acid	CD44 in CRC cells
	Yan et al. (2022) [32]	Supramolecular assembly	Cucurbit[7]uril	Spermine in CRC cells
	Chen et al. (2023) [33]	Bacterial membrane-fused liposomes	*F. nucleatum* membrane protein Fap-2	Gal-GalNAc overexpressed on CRC cells
	Chen et al. (2024) [34]	Bacterial membrane-fused liposomes	*F. nucleatum* membrane protein Fap-2	Gal-GalNAc overexpressed on CRC cells
	Zhuang et al. (2023) [36]	Biomimetic nanovaccine	*P. anaerobius* protein PCWBR2	α2β1 integrin on CRC cells
Phage therapy	Ding et al. (2025) [10]	Phage	Phage VA7	*B. fragilis*
	Dong et al. (2020) [42]	Phage–nanocomposites	M13 phage-displayed peptides	*F. nucleatum*

**Table 4:** Summary of biosafety assessments.

Category	Formulation/Targeting Strategy	Study	Biosafety Assessment Method
Passive targeting	Liposomes	Wang et al. (2024) [14]	In vivo: tumor-bearing mice Pharmacokinetics and Biodistribution Body weight Hematological and biochemical blood analysis H&E staining Gut microbiota analysis
	Liposomes	Wang et al. (2023) [23]	In vivo: tumor-bearing mice Body weight Body temperature Hematological analysis H&E staining
	Drug nanoassembly	Qiu et al. (2022) [24]	In vivo: CD-1 mice Pharmacokinetics and biodistribution Body weight Hematological analysis H&E staining
	Polymer	Li et al. (2024) [25]	In vitro 1. Mouse fibroblast cell line (L929) Cell viability 2. Hemolytic test
			In vivo: tumor-bearing mice Biodistribution Body weight H&E staining
	Inorganic	Chen et al. (2023) [26]	In vivo: male Kunming mice Body weight Hematological and biochemical blood analysis H&E staining
	Inorganic	Chu et al. (2022) [27]	In vitro 1. Human retinal epithelial cells (ARPE-19), African green monkey kidney cells (COS-7) and embryonic kidney cells (HEK-293T) Cell viability Cell cycle analysis ROS generation detection 2. Hemolytic test
			In vivo: tumor-bearing mice Body weight Biochemical blood analysis H&E staining Masson’s trichrome staining
	Drug nanoassembly	Gao et al. (2023) [28]	In vivo: tumor-bearing mice Biodistribution Body weight Biochemical blood analysis H&E staining Gut microbiota analysis
Active targeting	Targeting bacteria	Song et al. (2022) [29]	In vitro 1. Mouse embryonic fibroblasts (3T3) Cell viability 2. Hemolytic test
			In vivo: BALB/C mice Pharmacokinetics and biodistribution Body weight Hematological and biochemical blood analysis
	Targeting tumor	Li et al. (2023) [30]	In vitro (1) Mouse fibroblast cells (L929): cell viability (2) No CFU difference in *C. butyricum*, limited toxicity to *E. coli*
			In vivo: tumor-bearing mice Biodistribution Body weight
	Targeting tumor	Xie et al. (2024) [31]	In vitro 1. Mouse embryonic fibroblasts (NIH/3T3), human umbilical vein endothelial cells (HUVECs) Cell viability 2. Hemolytic test
			In vivo: tumor-bearing mice Pharmacokinetic and biodistribution Body weight Hematological and biochemical blood analysis H&E staining
	Targeting tumor	Yan et al. (2022) [32]	In vitro: normal human colon mucosal epithelial cell line (NCM460), mouse fibroblast cell line (L929) Cell viability
			In vivo 1. Female Wistar rats Pharmacokinetics 2. Male BALB/c nude mice Biodistribution Body weight H&E staining Survival rate
	Bacterial membrane-mimicking	Chen et al. (2023) [33]	In vivo: tumor-bearing mice Pharmacokinetics and biodistribution Gut microbiota analysis
	Bacterial membrane-mimicking	Chen et al. (2024) [34]	In vivo: tumor-bearing mice Biodistribution H&E staining
	Bacterial membrane-mimicking	Han et al. (2023) [35]	In vivo: tumor-bearing mice Biodistribution Hematological and biochemical blood analysis Immunofluorescent staining of biomarkers of macrophage and neutrophil Inflammatory cytokines concentration Body weight H&E staining Local lung microbiota diversity analysis
	Bacterial membrane-mimicking	Zhuang et al. (2023) [36]	In vitro: fetal colon epithelial cells (FHC) Cell viability In vivo: tumor-bearing mice Biodistribution Biochemical blood analysis H&E staining Gut microbiota analysis
Phototherapy	PDT	Liu et al. (2024) [37]	In vivo: tumor-bearing mice Biodistribution Body weight Biochemical blood analysis H&E staining Gut *E. coli*
	PTT	Kong et al. (2022) [38]	In vivo: female Kunming mice Pharmacokinetics and biodistribution Body weight Hematological and biochemical blood analysis H&E staining
	PTT	Kang et al. (2022) [39]	In vitro 1. Mouse fibroblast cell line (L929) Cell viability 2. Hemolytic test
			In vivo: male C57BL/6 mice Biodistribution Body weight Biochemical blood analysis H&E staining
Phage therapy	Phage	Ding et al. (2025) [10]	In vivo: male C57BL/6J mice, AOM/DSS-treated (model 2) Body weight Biochemical blood analysis
	Phage	Lam et al. (2025) [40]	In vivo: male BALB/c mice Body weight H&E staining
	Phage–nanocomposite	Zheng et al. (2019) [41]	In vivo 1. Bama minipiglets (*Sus scrofa*) Biodistribution Hematological and biochemical blood analysis 2. Tumor-bearing mice Body weight H&E staining Metabolites analysis Gut microbiota analysis
	Phage–nanocomposite	Dong et al. (2020) [42]	In vitro 1. M13 phage No PFU change under AgNP treatment 2. Mouse embryonic fibroblasts (3T3) Cell viability 3. Hemolytic test
			In vivo: female BALB/c mice Biodistribution Body weight Biochemical blood analysis H&E staining Fecal microbiota analysis
	Phage–nanocomposite	Zhao et al. (2025) [43]	In vitro: CT26 cells Cell viability ROS scavenging by DCFH-DA staining
			In vivo: male C57BL/6J mice (heathy and inflammatory bowel disease mice) Hematological and biochemical blood analysis Biodistribution in gastrointestinal tracts H&E staining Gut microbiota analysis
Other	Nanozyme	Wang et al. (2023) [44]	In vitro: normal human colon mucosal epithelial cell (NCM460), proximal tubule epithelial cell (HK-2) Cell viability
			In vivo: tumor-bearing mice Pharmacokinetics and biodistribution Body weight Hematological and biochemical blood analysis
	SDT	Qu et al. (2023) [45]	In vitro 1. Normal human colon mucosal epithelial cell line (NCM460): cell viability 2. Human keratinocyte (HaCaT) Cell viability, ROS, Calcein-AM/PI staining 3. Hemolysis
			Ex vivo Skin weight
			In vivo 1. Male BALB/c nude mice Pharmacokinetics and biodistribution Body weight Hematological and biochemical blood analysis H&E staining 2. Male C57BL/6 mice H&E staining

## Data Availability

No new data were created in this review. The protocol for this scoping review was retrospectively registered on OSF on 27 February 2026 (registration DOI: 10.17605/OSF.IO/K6BQR).

## Abbreviations

The following abbreviations are used in this manuscript:

^18^F-FDG-PET/CT	^18^F-fluorodeoxyglucose positron emission tomography/computed tomography
5-FU	5-fluorouracil
ACQ	Aggregation-induced quenching
AIE	Aggregation-induced emission
ALT	Alanine transaminase
AOM	Azoxymethane
APCs	Antigen-presenting cells
AST	Aspartate transaminase
BSA	Bovine serum albumin
CAC	Colitis-associated cancer
CFUs	Colony-forming units
CMF	Cyclophosphamide (CTX) + methotrexate (MTX) + 5-fluorouracil (5-FU)
CP	Capsular polysaccharide
CRC	Colorectal cancer
CREA	Creatinine
CTX	Cyclophosphamide
DBCO	Dibenzocyclooctyne
DMSNs	Dendritic mesoporous silica nanoparticles
DOX	Doxorubicin
DSS	Dextran sodium sulfate
EMA	European Medicines Agency
EMT	Epithelial–mesenchymal transition
EPI	Epirubicin
EPR	Enhanced permeability and retention
FDA	Food and Drug Administration
FDU	Fluorouridine
FISH	Fluorescence in situ hybridization
FOLFIRINOX	Folinic acid (leucovorin) + fluorouracil (5-FU) + irinotecan + oxaliplatin
FOLFOX	Folinic acid (leucovorin) + fluorouracil (5-FU) + oxaliplatin
GSH	Glutathione
HA	Hyaluronic acid
H&E	Hematoxylin–eosin staining
IBD	Inflammatory bowel disease
IHC	Immunohistochemistry
IRT	Irinotecan
i.g.	Intragastric
i.t.	Intratumoral
i.v.	Intravenous
LA	Lauric acid
LPS	Lipopolysaccharide
LTA	Lipoteichoic acid
Luc	Luciferase
MDSCs	Myeloid-derived suppressor cells
MET	Metronidazole
MTI	Metronidazole
MON	Metal–organic network
MSN	Mesoporous silica nanoparticles
MTX	Methotrexate
NIR	Near-infrared
NK	Natural killer cells
NPs	Nanoparticles
OEI	Oligomethyleneimine
OSF	Open Science Framework
OXA	Oxaliplatin
PBA	Phenylboric acid
PDT	Photodynamic therapy
Ppa	Pyropheophorbide-a
PTT	Photothermal therapy
PVP	Polyvinyl pyrrolidone
ROS	Reactive oxygen species
RT–qPCR	Reverse transcription quantitative polymerase chain reaction
SDT	Sonodynamic therapy
*Sg*	*Streptococcus gallolyticus*
s.c.	Subcutaneous
TAMs	Tumor-associated macrophages
TME	Tumor microenvironment
Treg	Regulatory T cells
ZIF	Zinc-imidazolate frameworks
